# Swarm Intelligence-Guided Hybrid Transfer Learning for Gastrointestinal Polyp Classification

**DOI:** 10.3390/biomimetics11080541

**Published:** 2026-08-03

**Authors:** Una Tuba, Mladen Veinovic, Eva Tuba, Adis Alihodzic, Milan Tuba

**Affiliations:** 1 Singidunum University, 11000 Belgrade, Serbia; utuba@ieee.org (U.T.); mveinovic@singidunum.ac.rs (M.V.); tuba@ieee.org (M.T.); 2 Jozef Stefan Institute, 1000 Ljubljana, Slovenia; 3 D.R. Semmes School of Science, Trinity University, San Antonio, TX 78212, USA; 4 Faculty of Science, University of Sarajevo, 71000 Sarajevo, Bosnia and Herzegovina; adis.alihodzic@pmf.unsa.ba; 5 Sinergija University, 76300 Bijeljina, Bosnia and Herzegovina

**Keywords:** gastrointestinal polyp classification, swarm intelligence, metaheuristic optimization, multi-backbone feature fusion, transfer learning, explainable AI, Grad-CAM, convolutional neural networks, Kvasir, colorectal cancer

## Abstract

Colorectal cancer remains a leading cause of cancer-related mortality worldwide, with automated polyp classification from endoscopic images offering a promising avenue for improving early detection. Existing approaches rely on single convolutional neural network (CNN) backbones with manually designed classification heads, limiting both representational capacity and deployment flexibility. This paper presents a swarm intelligence-augmented multi-backbone deep learning framework for eight-class gastrointestinal lesion classification on the Kvasir benchmark. Four CNN backbones (ResNet50, DenseNet121, MobileNetV2, EfficientNetB3) are independently fine-tuned using a two-phase transfer learning protocol and their penultimate-layer features concatenated into a 5888-dimensional representation, reduced to 256 dimensions via PCA. Five swarm intelligence algorithms—Particle Swarm Optimization, Artificial Bee Colony, JADE, L-SHADE, and CMA-ES—are benchmarked on the classification head architecture search task; all independently converge to tanh activation, a consistent pattern across independently initialized algorithms that is suggestive of, though not conclusive evidence for, particular geometric properties of PCA-transformed deep feature spaces. The PSO-optimized single-layer head (284 units, tanh) outperforms a manually designed three-layer baseline by 0.75% while using 67% fewer parameters. SI-guided class weight optimization yields targeted F1 improvements on the two most clinically significant classes (polyps: +0.015, ulcerative-colitis: +0.013). The fixed-head classifier trained on fused four-backbone features achieves 91.08% accuracy on Kvasir v2 (multi-seed mean 91.47% ± 0.49 across nine converging seeds; one seed failed to converge and is disclosed rather than excluded), below end-to-end DenseNet121 (92.25%; Wilcoxon *p* = 0.31, not statistically significant), while enabling classifier updates in under 30 s; a three-backbone subset dropping the weakest backbone (EfficientNetB3) reaches 92.33%, exceeding the full four-backbone fusion. Cross-dataset evaluation on Kvasir v1-to-v2 confirms near-zero generalization gaps across dataset scales; a restricted two-class evaluation on HyperKvasir (the only two of eight classes with usable labeled data) reaches 96.28% accuracy, and dual Grad-CAM with SI minimal sufficient region analysis, validated quantitatively against Kvasir-SEG ground-truth masks, provides spatially grounded, clinically interpretable explanations.

## 1. Introduction

Colorectal cancer (CRC) kills over 900,000 people annually and ranks as the second leading cause of cancer-related death worldwide [1]. By 2040, incidence is projected to reach 3.2 million cases per year, up 66% from 2020 [2]. Most CRCs develop from gastrointestinal polyps—benign mucosal growths that can undergo malignant transformation over years if undetected. Colonoscopic removal of such lesions substantially reduces mortality [3], yet polyp miss rates remain at 20–25% even in expert hands, attributable to operator fatigue, suboptimal bowel preparation, and the subtle appearance of flat or diminutive lesions [4]. Automated, computer-aided classification of endoscopic images therefore addresses a genuine and persistent clinical need.

CNNs have become the standard tool for medical image classification [5], and transfer learning from ImageNet now dominates how researchers adapt pretrained representations to limited clinical datasets [6]. On the Kvasir benchmark [7], Cristea et al. [8] achieved 92.3% accuracy using ResNet50, DenseNet121, and MobileNetV2—but reported only accuracy and per-class F1 on a single dataset, with no MCC, no AUC-ROC, no cross-dataset testing, and no statistical significance analysis. Hyperparameter choices were manual throughout. This is not unusual: across the literature, backbones tend to be trained in isolation rather than combining their complementary features, classification heads are designed by hand, and explainability is typically applied post hoc to a single model.

Swarm intelligence (SI) algorithms (nature-inspired metaheuristics such as Particle Swarm Optimization (PSO) [9] and Artificial Bee Colony (ABC) [10]) are well-suited to the non-convex, mixed discrete–continuous spaces that characterize neural architecture search, where gradient-based methods do not apply. Their use in medical imaging has so far focused on optimizing backbone architectures [11]. Applying SI to optimize lightweight classification heads over frozen deep features—a cheaper and more modular problem—has received little systematic attention.

Beyond its role as an optimizer, SI search here also functions as a diagnostic tool. Five independently initialized, structurally different SI algorithms converge on the same activation function when searching the classification head’s architecture space (Section 5.2); manual architecture design, tested across a handful of trials, would be unlikely to surface this kind of convergent signal. We treat this as a methodological illustration of what bio-inspired population search can reveal about a problem’s structure, rather than solely as a route to a marginally better accuracy number—the accuracy differences involved are modest, and we report them as such throughout.

This paper presents a swarm intelligence-augmented hybrid deep learning framework for gastrointestinal polyp classification that addresses the identified gaps through five contributions:

1. Multi-backbone feature fusion. Features from four independently fine-tuned CNN backbones (ResNet50, DenseNet121, MobileNetV2, EfficientNetB3) are extracted from frozen penultimate layers and concatenated into a unified 5888-dimensional representation. A shallow classifier trained on this fused representation performs comparably to the strongest single backbone (Section 5.3) while enabling modular classifier updates without backbone retraining; a subsequent ablation shows that dropping the weakest backbone from the fusion improves on the full four-backbone result (Section 5.3.1).
2. SI-guided classification head optimization. PSO, ABC, JADE, L-SHADE, and CMA-ES are benchmarked on the head architecture search problem, treating layer count, units, dropout, learning rate, activation, and batch size as a joint search space. All five algorithms independently converge to the same activation function (Section 5.2), and the winning architecture is substantially smaller than a manually designed baseline while matching or exceeding its accuracy (Section 5.4).
3. SI-guided class weight optimization. A secondary PSO run searches per-class training weights to maximize macro F1, targeting improvements on the two most clinically significant classes (Section 5.5).
4. Cross-dataset generalization. Models trained on Kvasir v1 are evaluated on Kvasir v2, and separately on a verified subset of HyperKvasir, establishing generalization behavior across both dataset scale and imaging source (Section 5.7).
5. Comprehensive evaluation and explainability. Performance is reported across accuracy, macro F1, MCC, and AUC-ROC with multi-seed evaluation and Wilcoxon signed-rank significance testing, extending the evaluation depth of the reference work. Grad-CAM and SI-driven minimal sufficient region search are validated quantitatively against expert-verified segmentation masks, rather than presented only qualitatively (Section 5.8).

The remainder of this paper is structured as follows. Section 2 reviews related work on CNN-based GI polyp classification and SI applications in medical imaging. Section 3 describes the proposed framework in detail. Section 4 presents the experimental setup. Section 5 reports and analyzes the results. Section 6 discusses the findings. Section 7 concludes with directions for future work.

## 2. Related Work

### 2.1. CNN-Based Gastrointestinal Lesion Classification

Deep convolutional neural networks are now the default approach for automated classification of GI endoscopy images. The Kvasir dataset [7] provides the standard eight-class benchmark for this task and forms the basis of the evaluation here.

Transfer learning from ImageNet-pretrained backbones is the established paradigm for limited clinical datasets. Cuevas-Rodriguez et al. [12] compared ResNet-50, Inception-V3, and VGG-16 across five combined colonoscopy databases, reaching 97% accuracy on pooled data but noting that cross-source generalization remains the primary difficulty. Cristea et al. [8] reached 92.3% on Kvasir v1 with three backbones, without MCC, AUC-ROC, cross-dataset evaluation, or principled hyperparameter search.

Ensemble methods improve on single-backbone results. Younas et al. [13] combined six CNN classifiers for three-class polyp subtype detection, reporting macro F1 alongside accuracy. Gunasekaran et al. [14] proposed GIT-Net, a weighted average of DenseNet201, InceptionV3, and ResNet50 on Kvasir v2, reaching 95% accuracy. Neither work optimizes ensemble weights automatically. Mukhtorov et al. [15] paired ResNet152 with Grad-CAM to achieve 93.46% validation accuracy with visual explanations, establishing that accuracy and interpretability should be pursued together.

Some recent results push well beyond these numbers. Bhardwaj et al. [16] reported 99.75% accuracy combining EfficientNetB6 and DenseNet169, but on a subset of Kvasir classes rather than the full eight-class taxonomy, and the result has not been independently replicated. Shen et al. [17] deployed EfficientNet-b0 across 256,220 colonoscopy images from multiple hospitals, with sensitivity and specificity above 0.97. That scale underscores how demanding real deployment is.

### 2.2. Swarm Intelligence for Neural Architecture Search and Hyperparameter Optimization

SI algorithms have been applied to CNN architecture search in medical imaging precisely because the search space is non-convex and mixed. Aguerchi et al. [18] used PSO to optimize kernel sizes, strides, and filter counts for mammographic classification, reaching 98.23% on DDSM; a practical observation from that work is that automated search removes the need for domain expertise in network design. Amoury et al. [19] similarly applied PSO to the full CNN architecture for MRI brain tumor classification, reaching 99.19%. Both papers optimize the backbone itself. Our contribution is different: we optimize only the classification head over frozen features, which is considerably cheaper and more compatible with modular deployment.

Yonar et al. [20] combined PSO and the Grey Wolf Optimizer for feature selection over multi-backbone representations using DenseNet201, MobileNetV2, ResNet50, and InceptionV3—a multi-backbone setup that parallels ours, though with feature selection rather than PCA-based reduction. Hamad et al. [21] surveyed metaheuristic CNN hyperparameter optimization across 2019–2022, concluding that population-based methods consistently outperform grid and random search. That conclusion motivates the five-algorithm comparison in Section 5.2. Xu et al. [22] surveyed SI applications to image processing in *Biomimetics*, identifying medical classification as underexplored for SI-based architecture search.

### 2.3. Explainability in Endoscopy AI

Explainability is increasingly treated as a prerequisite for clinical adoption rather than an optional add-on. Mukhtorov et al. [15] showed that Grad-CAM applied to ResNet152 on Kvasir produces attribution maps that improve clinician trust without hurting accuracy. Cox et al. [23] reviewed XAI for upper GI endoscopy and found class activation mapping to be the dominant method, though almost always applied post hoc to a single model. Sutton et al. [24] graded ulcerative colitis severity with DenseNet121 and Grad-CAM on HyperKvasir, reaching AUC 0.90 and showing that the model’s attention regions correspond to mucosal features that clinicians actually use.

The SI minimal sufficient region approach used here belongs to the perturbation-based XAI family [21]: rather than asking which gradients are large, it asks what is the smallest image crop that preserves the model’s prediction. This has been applied in general medical image analysis [25] but not to GI endoscopy specifically.

### 2.4. Gaps Addressed by This Work

This manuscript’s specific contribution, relative to the work reviewed above, is fourfold. First, we apply SI to optimize classifier head architecture and per-class training weights together on Kvasir, and evaluate cross-dataset generalization for the resulting pipeline; we do not claim this combination is unique across the broader literature, only that it has not been applied to this dataset and task before. Second, our SI architecture search targets a lightweight head over frozen multi-backbone features rather than full-backbone optimization, which is the more common target of SI-based architecture search in medical imaging (Section 2.2); this is a narrower and cheaper search problem, not a claim of general superiority over full-backbone search. Third, we combine multi-backbone Grad-CAM with a SI minimal region search for this specific classification task, which to our knowledge has not been done together in GI endoscopy before. Fourth, we report MCC and AUC-ROC alongside accuracy and per-class F1, which the reference work of Cristea et al. [8] does not.

## 3. Proposed Method

**Figure 1:**
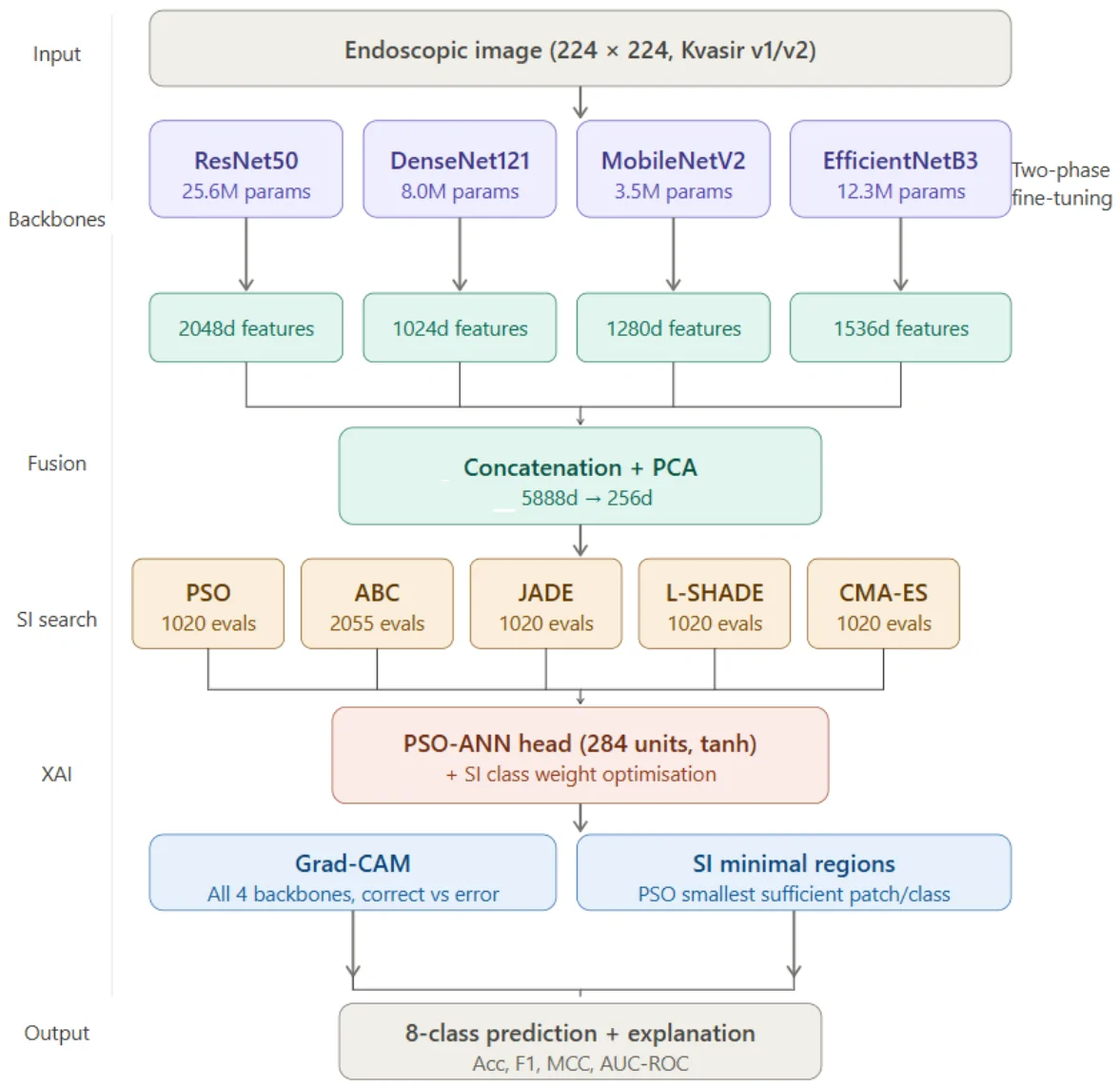
Schematized proposed framework.

The framework has four sequential stages (Figure 1): backbone fine-tuning, feature extraction and fusion, SI-guided head architecture search, and SI-guided class weight optimization. Stages 3 and 4 operate on frozen features, so the classifier can be updated without retraining the backbones.

### 3.1. Transfer Learning and CNN Backbone Fine-Tuning

Starting from ImageNet weights [26] rather than random initialization lets the model reuse low-level visual representations learned from millions of natural images [6]. Given the limited size of annotated endoscopy datasets, this is not merely convenient but practically necessary.

Four CNN architectures were selected to provide complementary inductive biases and feature extraction characteristics:

ResNet50 [27] (25.6 M parameters) uses residual skip connections, producing representations that combine low-level texture with high-level semantic abstractions. DenseNet121 [28] (8.0 M parameters) connects each layer to all subsequent layers, promoting feature reuse and fine-grained texture discrimination well-suited to endoscopic images. MobileNetV2 [29] (3.5 M parameters) uses depthwise separable convolutions for computational efficiency, making it suitable for resource-constrained deployment. EfficientNetB3 [30] (12.3 M parameters) scales depth, width, and resolution jointly, achieving strong feature extraction with fewer floating-point operations than ResNet or DenseNet variants of comparable accuracy. Together, the four architectures cover a spectrum from residual connectivity to dense reuse, efficient separability, and compound scaling, ensuring that the fused feature space captures complementary visual information.

#### 3.1.1. Two-Phase Fine-Tuning Protocol

Full fine-tuning from the start risks catastrophic forgetting: gradients from an untrained classification head can corrupt pretrained weights before the head has learned anything useful. A two-phase protocol addresses this.

Phase 1 freezes the entire backbone and trains only the classification head for five epochs at $10^{-3}$. This stabilizes the head weights before any backbone parameters change.

Phase 2 unfreezes the top 50% of backbone layers (preserving general low-level detectors in the lower half) and fine-tunes at $10^{-4}$ for up to 40 epochs, with early stopping (patience = 8) and learning rate reduction on plateau (factor = 0.5, minimum $10^{-7}$). The reduced rate prevents the large gradient steps that would otherwise overwrite pretrained representations.

#### 3.1.2. Classification Head Architecture

Each backbone is augmented with an identical classification head: global average pooling (GAP) is applied to the final convolutional feature map, collapsing spatial dimensions while retaining channel-wise statistics. GAP is followed by batch normalization, a dropout layer (rate = 0.4), a dense layer of 256 units with ReLU activation, a second dropout layer (rate = 0.2), and a softmax output layer of K=8 units corresponding to the Kvasir class taxonomy. All models are trained with the Adam optimizer [31] and sparse categorical cross-entropy loss. Class-weighted sampling is applied during training to compensate for residual class imbalance.

### 3.2. Multi-Backbone Feature Fusion

After training, each backbone is frozen and used purely as a feature extractor. The global average pooling output (the penultimate representation before the classification head) is extracted for every image in the training, validation, and test sets. These vectors capture the learned visual representation without the backbone-specific classification bias.

Vectors from all four backbones are concatenated:

(1) $$f=[f_{\text{ResNet50}}\;∥\;f_{\text{DenseNet121}}\;∥\;f_{\text{MobileNetV2}}\;∥\;f_{\text{EfficientNetB3}}]$$

giving a 5888-dimensional vector (2048+1024+1280+1536). The concatenation combines features from four architecturally distinct families: residual skip connections, dense layer connectivity, efficient separable convolutions, and compound scaling.

PCA is then applied to the standardized vectors, retaining 256 components. This preserves approximately 83.5% of training-set variance while reducing dimensionality by a factor of 23. A larger number of components would retain more variance but was not pursued here, since the classifier head architecture search (Section 3.3) was tuned against this 256-dimensional input. A StandardScaler and PCA transform are fitted on training features only and applied to validation and test sets, so there is no leakage. The practical payoff of this modular design is that the shallow classifier can be retrained from scratch in seconds when new data arrives, without touching the backbone weights.

### 3.3. Swarm Intelligence Algorithms

SI algorithms are population-based metaheuristics suited to non-convex, mixed discrete–continuous problems with no differentiable objective—all characteristics of neural architecture search. A population of candidate solutions is maintained and updated iteratively, balancing exploration of unseen regions against exploitation of known good solutions.

Grid search does not scale to the ten-dimensional mixed discrete–continuous space in Table 1, and random search provides no mechanism for exploiting promising regions once found. Bayesian optimization is a plausible alternative for the continuous hyperparameters, but handles the mixed discrete choices (number of layers, activation function, batch size) less naturally than population-based metaheuristics, which treat the encoding uniformly. SI algorithms were chosen for this reason, not because alternatives were tested and found inferior; a direct comparison against Bayesian optimization on this search space is a reasonable direction for future work.

#### 3.3.1. Algorithms Used

Five algorithms are benchmarked, selected to cover a broad spectrum of search mechanisms. **PSO** [9] models particles moving through the search space, each updating its velocity according to its personal best and the swarm’s global best:

(2) $$v_{i}\leftarrow wv_{i}+c_{1}r_{1}\left(p_{i}-x_{i}\right)+c_{2}r_{2}(g-x_{i}),\;x_{i}\leftarrow x_{i}+v_{i}$$

where *w* is an inertia weight, $c_{1}$, $c_{2}$ are cognitive and social acceleration coefficients, $r_{1},r_{2}∼U\left(0,1\right)$, $p_{i}$ is the personal best, and g is the global best. PSO is parameter-efficient and converges rapidly on smooth landscapes. **ABC** [10] partitions search effort among employed, onlooker, and scout bee roles, achieving strong exploration at the cost of more fitness evaluations. **JADE** [32] and **L-SHADE** [33] are self-adaptive Differential Evolution [34] variants that adapt crossover and scaling parameters from successful mutations; L-SHADE additionally reduces population size linearly to concentrate budget on exploitation. **CMA-ES** [35] maintains a multivariate Gaussian over the search space and adapts its full covariance matrix, making it the standard benchmark for continuous black-box optimization on ill-conditioned problems.

#### 3.3.2. Architecture Search Formulation

The classification head architecture search is formulated as a minimization problem over a ten-dimensional mixed search space $X\subset R^{10}$:

(3) $$x^{*}=\arg\min_{x\in X}\;f\left(x\right)=1-\mathrm{val}\_\mathrm{acc}\left(\phi \left(x\right)\right)$$

where ϕ(x) denotes the MLP classifier parameterized by the decoded solution vector x, and val_acc(·) is the accuracy on the held-out validation set after training for 15 epochs. The search space dimensions are defined in Table 1.

The fitness function evaluates real classifier performance on the actual PCA-reduced feature space, requiring no surrogate proxy model. Each evaluation trains a candidate MLP for 15 epochs with early stopping (patience = 5), returning the best validation accuracy achieved. This surrogate-free approach ensures that the fitness landscape reflects the true objective, at the cost of approximately 0.5–2 s per evaluation on CPU. All five algorithms use population size 20 and a maximum of 50 epochs, yielding 1000–2055 fitness evaluations per algorithm.

### 3.4. SI-Guided Class Weight Optimization

Class imbalance in training data causes classifiers to favor majority classes, reducing recall on clinically important minority classes such as polyps and esophagitis. While standard approaches assign class weights inversely proportional to class frequency, these heuristic weights may not optimize the metric of primary clinical interest. We formulate class weight selection as a secondary PSO optimization problem:

(4) $$w^{*}=\arg\max_{w\in {\left[0.1,10\right]}^{K}}\;{F1}_{\mathrm{macro}}\left(\phi^{*};w\right)$$

where $\phi^{*}$ is the SI-optimized classifier, K=8 is the number of classes, and ${F1}_{\mathrm{macro}}\left(\phi^{*};w\right)$ is the macro-averaged F1 score on the validation set when the training set is oversampled according to weights w. Oversampling is implemented by resampling each class to $\max(\left\lfloor n_{k}\cdot w_{k}\right\rfloor ,n_{k})$ samples with replacement, where $n_{k}$ is the original class count. This formulation allows PSO to discover class emphasis patterns that differ from frequency-based heuristics, directly optimizing the evaluation metric of interest.

### 3.5. Explainability

High accuracy alone is not sufficient for clinical deployment. Two complementary approaches are used to explain individual predictions.

Grad-CAM [36] weights the final convolutional feature map channels by their globally averaged gradient magnitudes with respect to the class score, producing a spatial importance map. Applied independently to all four backbones, this yields four attribution maps per image. Comparing correct and misclassified predictions reveals which regions systematically drive errors.

SI minimal sufficient region search uses PSO to find the smallest bounding box [x,y,w,h] that, when cropped and classified, still maintains the model’s confidence for the correct class. The fitness function is

(5) $$f\left(x,y,w,h\right)=-\left[p_{c}\left(x,y,w,h\right)\cdot \left(1-\frac{w\cdot h}{H\cdot W}\right)\right]$$

where $p_{c}$ is the correct-class probability on the crop and H×W is the full image area. This gives a lower bound on how much of the image the model actually needs. Like Grad-CAM, this is a perturbation-based, correlational measure rather than a causal one; it offers a complementary, sufficiency-based view of what the classifier relies on, not a stronger causal claim.

## 4. Experimental Setup

### 4.1. Datasets

Experiments were conducted on two publicly available gastrointestinal endoscopy datasets. The primary benchmark was Kvasir v2 [7], comprising 8000 images across eight classes: dyed-lifted-polyps, dyed-resection-margins, esophagitis, normal-cecum, normal-pylorus, normal-z-line, polyps, and ulcerative-colitis, with 1000 images per class. For direct comparison with the reference work of Cristea et al. [8], experiments were also conducted on Kvasir v1, which contains 4000 images (500 per class) covering the same eight classes. Cross-dataset generalization was evaluated on HyperKvasir [37], a labeled dataset of approximately 10,000 images across 23 classes, of which eight overlap with Kvasir’s taxonomy in principle; however, usable labeled data was only obtained for two of those eight classes (dyed-lifted-polyps and dyed-resection-margins, n = 1991 combined), and the evaluation in Section 5 is restricted accordingly.

All datasets were partitioned using stratified sampling into training (70%), validation (15%), and test (15%) subsets, preserving class distributions across splits. For Kvasir v2, this yielded 5600 training, 1200 validation, and 1200 test images. Images were resized to 224×224 pixels and normalized to [0,1].

### 4.2. Model Training and Search Configuration

All backbone training, feature fusion, SI architecture search, and class weight optimization followed the protocols described in Section 3. Backbone training used ImageNet-pretrained weights with the two-phase protocol (Phase 1: 5 epochs at $10^{-3}$; Phase 2: up to 40 epochs at $10^{-4}$ with early stopping). SI searches used a population size of 20 and a maximum of 50 epochs. All experiments used a fixed random seed (42) for reproducibility. Implementations used TensorFlow 2.19, scikit-learn 1.8, and mealpy 3.0.3.

### 4.3. Computational Environment

Experiments were conducted on Google Colab Pro using NVIDIA A100 (40 GB) and T4 (16 GB) GPUs. Backbone training required approximately 40–60 min per model on A100. Feature extraction, PCA, and classifier training completed in under 30 min. SI architecture search required approximately 35–94 min per algorithm depending on convergence (PSO: 35.9 min, ABC: 93.8 min, JADE: 41.1 min, L-SHADE: 38.0 min, CMA-ES: 56.0 min). Total pipeline runtime from raw data to final results was approximately 8–10 h.

## 5. Results

Results are presented in the order of the pipeline. Table 2 provides a structured overview of each subsection and its key finding.

### 5.1. CNN Backbone Fine-Tuning Performance

Table 3 shows test set performance on the 1200-image Kvasir v2 held-out split. DenseNet121 leads across all four metrics: 92.25% accuracy, F1 0.9224, MCC 0.9117, AUC-ROC 0.9947. MobileNetV2 reaches 90.42% with a notably high AUC-ROC (0.9927), suggesting well-calibrated probabilities even where raw accuracy is lower. ResNet50 and EfficientNetB3 both fall near 81%, substantially behind the other two; their MCC values (0.810 and 0.807) reflect higher confusion on visually ambiguous classes.

**Figure 2:**
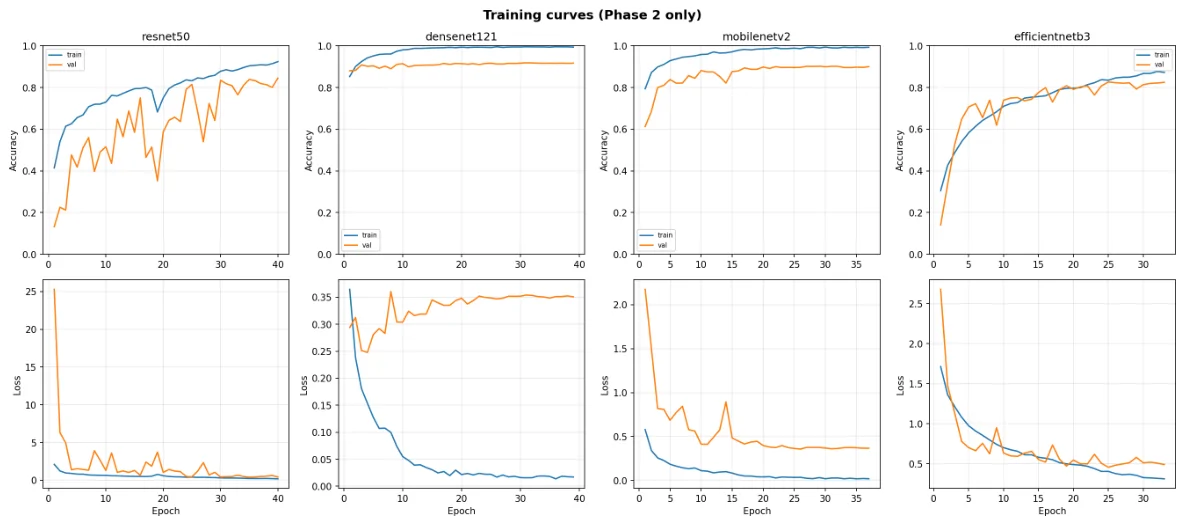
Training and validation accuracy/loss curves for all four CNN backbones under the two-phase fine-tuning protocol. Phase 1 (5 epochs, frozen backbone) and Phase 2 (fine-tuning) are visually distinguishable by the change in learning dynamics.

Training curves (Figure 2) show the expected two-phase pattern: a sharp accuracy rise in Phase 1 as the head learns from frozen features, then a slower improvement in Phase 2 as the upper backbone layers adapt. DenseNet121 and MobileNetV2 converge with tight training–validation gaps. ResNet50 and EfficientNetB3 are noisier throughout. EfficientNetB3’s underperformance relative to its ImageNet benchmark is worth noting: its compound-scaled architecture targets 300×300 input, and the fixed 50% unfreeze strategy does not match EfficientNet’s uniform depth-width-resolution scaling the way it matches ResNet or DenseNet.

**Figure 3:**
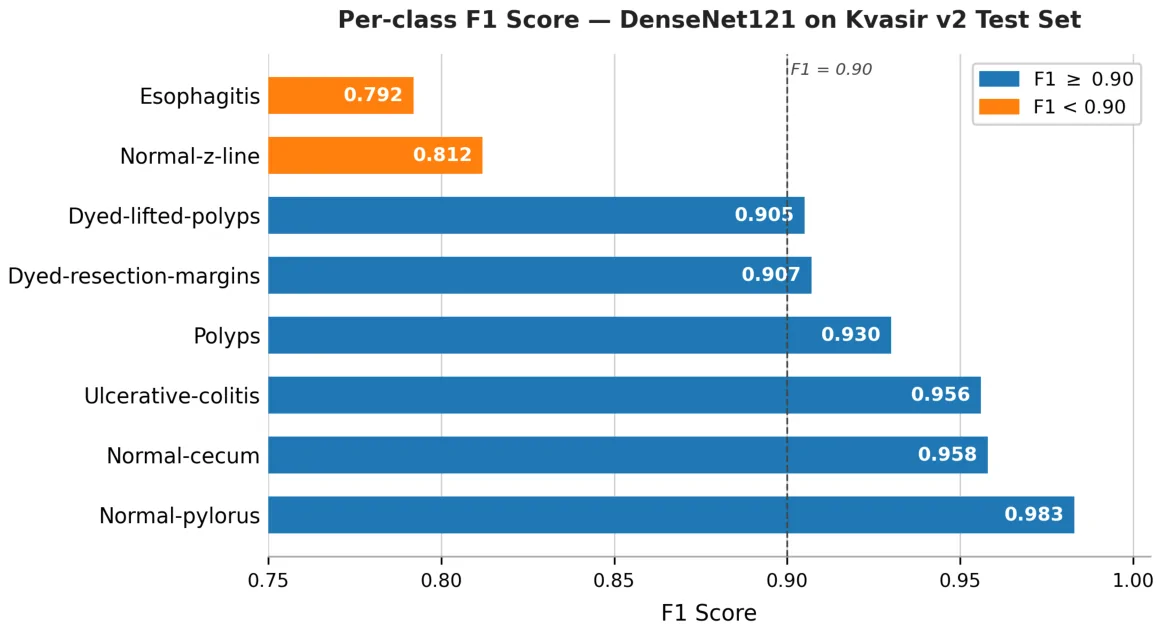
Per-class F1 scores for DenseNet121 on the Kvasir v2 test set, ordered by performance. Esophagitis and z-line are consistently the weakest classes across all backbones.

Per-class F1 for DenseNet121 (Figure 3) is telling. Pylorus (0.983) and cecum (0.958) are easy—their visual signatures are distinctive and well-captured by ImageNet pretraining. Ulcerative-colitis (0.956) and polyps (0.930) also score well. Esophagitis (0.845) and normal-z-line (0.855) are consistently the weakest pair across all backbones, a pattern that drives the class weight optimization in Section 5.5.

### 5.2. SI Algorithm Benchmarking

Table 4 shows results for all five algorithms under identical conditions (population 20, maximum 50 epochs, same PCA-reduced features; fitness: 15-epoch MLP returning 1−val_accuracy).

Every algorithm converges to tanh, starting from random initialization in a space that includes relu and elu. This consistent convergence across five independently initialized algorithms is a suggestive pattern rather than a proven property of the feature space. One plausible explanation is that PCA-transformed CNN features sit on a compact, bounded manifold where a saturating nonlinearity separates classes more reliably than unbounded relu, but we did not test this geometric hypothesis directly, and other explanations cannot be ruled out.

PSO reaches 0.9242 in 1020 evaluations and 35.9 min—the best result in the fewest calls. ABC matches the accuracy but needs 2055 evaluations, more than double, owing to its role-partitioned foraging: ABC’s employed, onlooker, and scout bee roles each contribute separate evaluations per epoch, so at the same population size and epoch budget it inherently produces more fitness evaluations than PSO or the Differential Evolution variants. This is an intrinsic property of the algorithm’s structure rather than an inconsistency in how the comparison was configured, and we report the resulting evaluation counts in Table 4 transparently rather than holding them artificially equal across algorithms. JADE, L-SHADE, and CMA-ES each reach 0.9233, within 0.001 of the top. Near-optimal solutions are apparently not rare in this search space.

**Figure 4:**
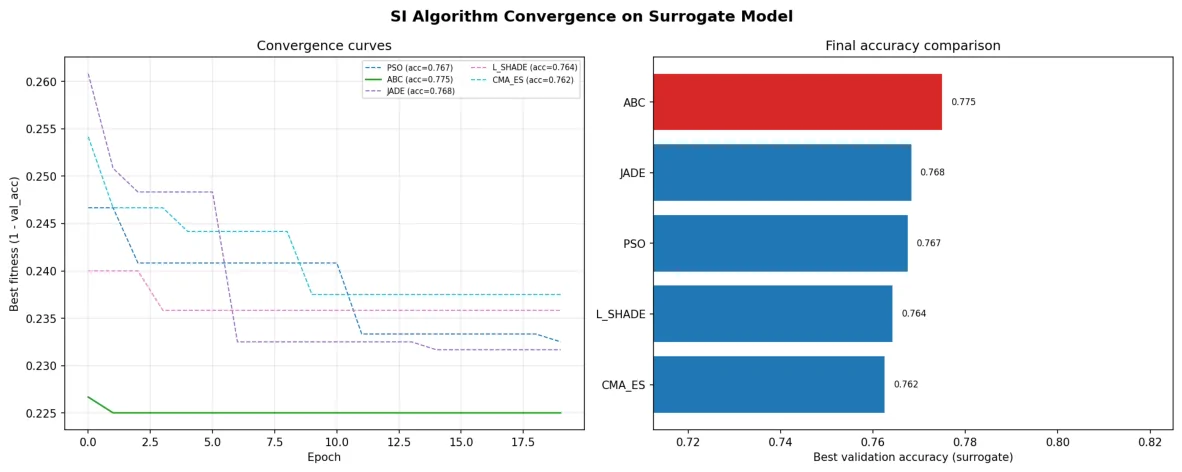
Convergence curves for all five SI algorithms on the ANN head architecture search task. Lower fitness = higher validation accuracy. PSO converges fastest; all algorithms converge to tanh activation.

Figure 4 shows how each algorithm converges. PSO reaches near-optimal fitness within roughly 15 epochs. CMA-ES descends smoothly as its covariance matrix narrows. L-SHADE and JADE track each other closely; ABC is slow early, then improves sharply once employed bees focus on the best regions.

PSO is selected as the winning algorithm. Its architecture (a single hidden layer of 284 units, *tanh* activation, learning rate $3.74\times 10^{-3}$, batch size 64) is used for full training in Section 5.4. The surrogate faithfulness of the 15-epoch fitness function is verified in Section 5.4: the surrogate validation accuracy of 0.9242 correctly ranks PSO above the manual baseline, confirming that the 15-epoch proxy provides a reliable ranking signal despite its brevity.

### 5.3. Multi-Backbone Feature Fusion

Table 5 compares ANN classifiers trained on single-backbone features against the concatenated four-backbone representation, with end-to-end DenseNet121 as an external reference. All ANN classifiers use the fixed 512–256–128 baseline trained for 500 epochs, so performance differences reflect the input representation, not the classifier.

Fusing all four backbones gives 0.9108 accuracy—+0.005 above the best single-backbone ANN (DenseNet121 features, 0.9058) and +0.024 above MobileNetV2 features. The gain is consistent across accuracy, F1, and MCC, so it is not driven by majority classes alone. DenseNet121 captures fine-grained texture; ResNet50 captures broader structure; neither alone produces what the combination does.

The fused ANN (0.9108) does not exceed end-to-end DenseNet121 (0.9225). The practical case for the fused representation is operational rather than accuracy-based: the classifier can be retrained from scratch in seconds when new data arrives, versus 40–60 min of GPU time to retrain DenseNet121 end-to-end, at a modest accuracy cost relative to the strongest single backbone. Section 5.6 additionally shows that dropping the weakest backbone (EfficientNetB3) from the fusion improves on this result further.

**Figure 5:**
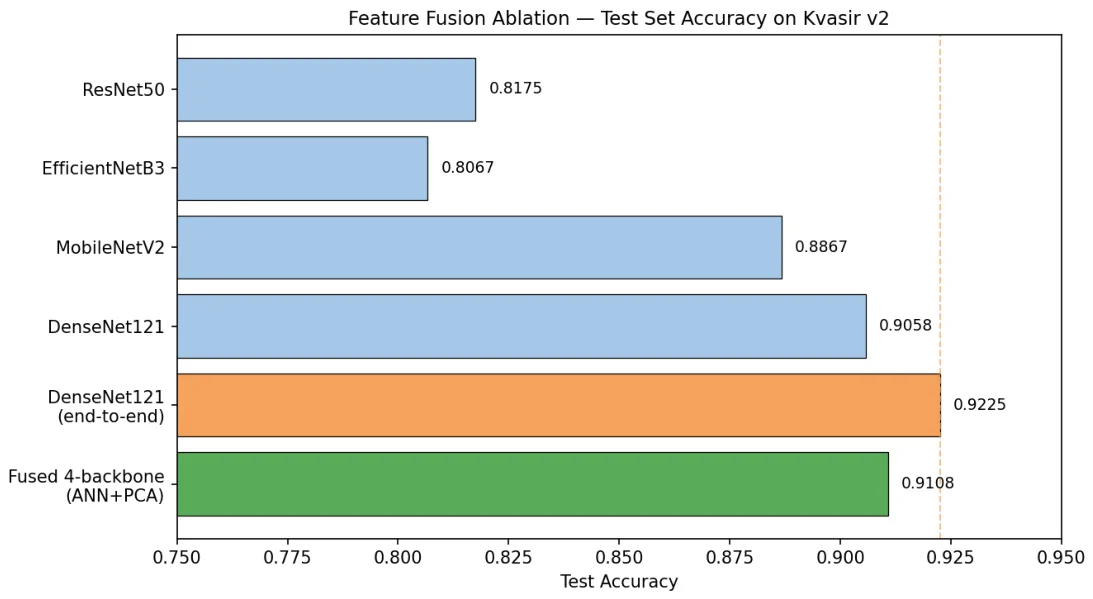
Test set accuracy for ANN classifiers trained on individual backbone feature vectors vs. the concatenated four-backbone representation (after PCA to 256d). The dashed line marks the end-to-end DenseNet121 baseline.

PCA to 256 components retains 83.5% of training variance and acts as a mild regularizer—low-variance directions likely encode noise rather than useful signal. Figure 5 shows the stepwise accuracy improvement from single backbone to fused representation.

#### 5.3.1. Backbone Subset Ablation

The four-backbone fusion in Table 5 raises a natural question: are all four backbones necessary, or would a subset perform as well or better? Table 6 reports accuracy for every two-backbone and three-backbone subset of the four CNN backbones, evaluated under the same standardized protocol (256-component PCA, both the fixed baseline and PSO-ANN architectures).

The best-performing subset for both architectures is ResNet50 + DenseNet121 + MobileNetV2—three backbones, dropping EfficientNetB3—reaching 0.9192 (fixed baseline) and 0.9233 (PSO-ANN), both higher than the full four-backbone fusion (0.9108 and 0.9183 respectively). This is consistent with EfficientNetB3 being the weakest individual backbone by end-to-end classification accuracy (Table 3, 0.8075): including its features in the fusion appears to add noise rather than complementary signal. Every two-backbone subset that excludes both ResNet50 and EfficientNetB3 (i.e., DenseNet121 + MobileNetV2) also outperforms several three-backbone subsets that include one of the weaker backbones, reinforcing that backbone quality, not backbone count, drives fusion performance. All four backbones are therefore not necessary for this fusion to be effective; a three-backbone subset excluding the weakest classifier is both cheaper to compute and more accurate.

### 5.4. SI-Optimized Classification Head

Given the fused features, does PSO-guided search actually improve the classifier over a manually designed head? To test this fairly, both architectures are trained for 500 epochs with early stopping (patience = 15) on identical PCA-reduced inputs. The only difference is the head.

PSO-ANN reaches 0.9183, beating the manual baseline by +0.0075 (Table 7). The gain holds for F1 macro (+0.0070). More striking is the parameter count: 73K versus 220K—a 67% reduction. The manual three-layer design was simply overparameterized for a 256-dimensional input.

The surrogate ranking also holds: the 15-epoch proxy correctly places PSO-ANN above the baseline, and that ordering survives full training under a standardized protocol. The PSO search (1020 evaluations, 35.9 min) cost about as much as training one backbone from scratch. That is a reasonable price for automated architecture selection.

A single layer outperforming three is not surprising once the input dimensionality is considered. After PCA to 256 components the problem is much simpler than raw pixel classification, and deeper networks add optimization complexity with no representational payoff. The *tanh* result from Section 5.2 reinforces this: the bounded PCA manifold suits saturating nonlinearities.

The PSO-ANN and the fixed baseline are both evaluated under the identical protocol described in Section 3 (256-component PCA, 500-epoch training budget with early stopping, patience 20).

**Figure 6:**
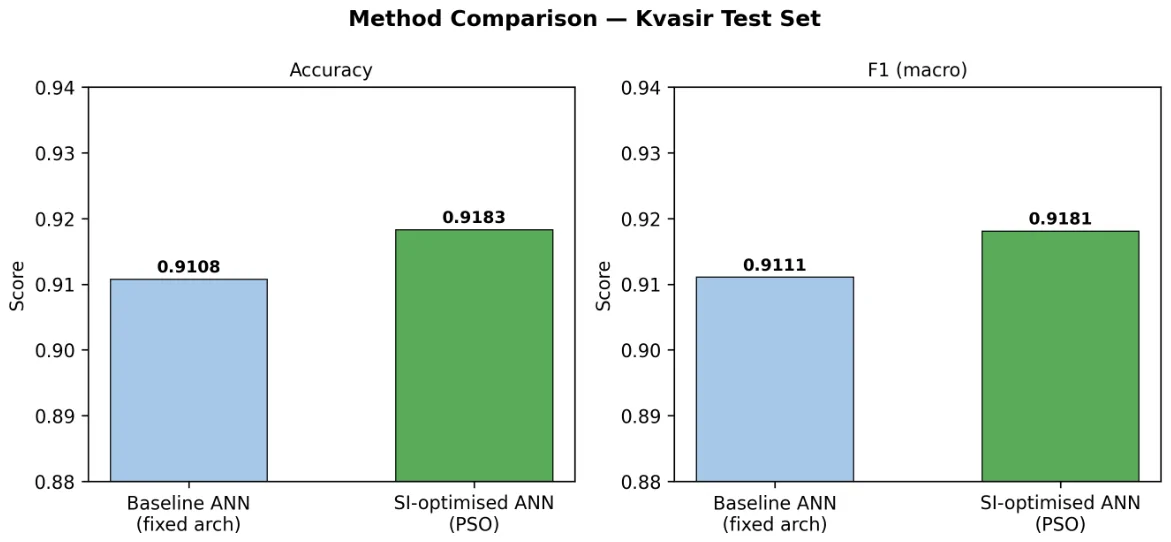
Test set accuracy of the manually designed three-layer baseline vs. the PSO-optimized single-layer head under a fair 500-epoch training budget on identical PCA-reduced features.

**Figure 7:**
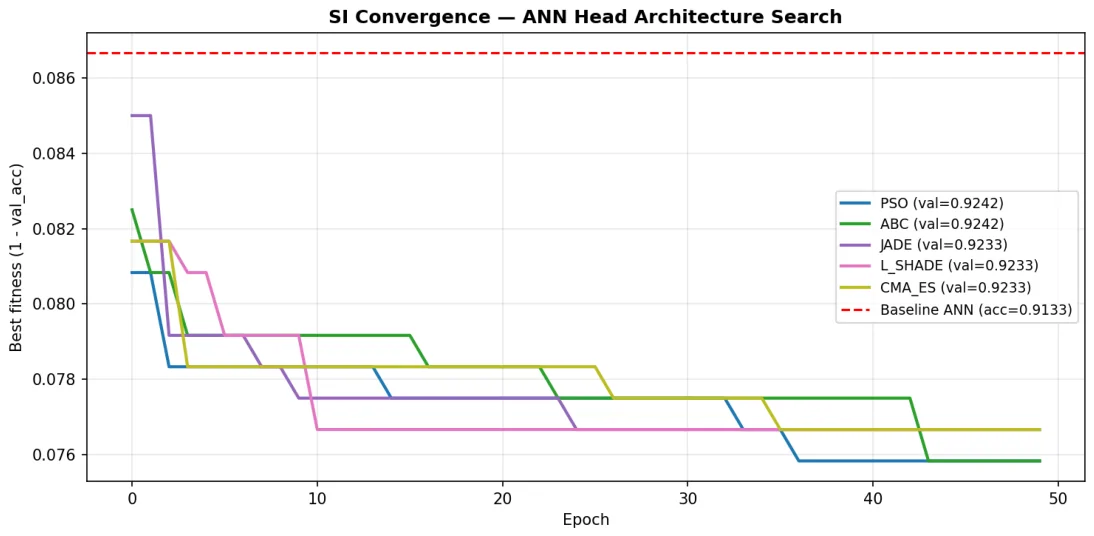
PSO convergence during ANN head architecture search (1020 evaluations, population 20, 50 epochs). The fitness plateaus after approximately 15 epochs, consistent with the surrogate faithfulness result.

Figure 6 compares accuracy and F1-score on the test set for the baseline ANN and the SI-optimized ANN. Figure 7 shows convergence curves for all five SI algorithms that have been tested.

### 5.5. SI-Guided Class Weight Optimization

Training the PSO-ANN head with uniform class weights leaves a residual performance gap on the two weakest Kvasir classes, esophagitis and normal-z-line, which have consistently lower per-class F1 scores across all backbones (Section 5.1). To address this, a secondary PSO run searches for per-class loss weights $w\in R_{>0}^{8}$ that maximize macro F1 on the validation set, using the trained PSO-ANN head as the fixed classifier and reweighting only the training loss. This decouples weight optimization from architecture search and adds no inference-time overhead.

Table 8 reports the SI-optimized weights alongside the per-class F1 of the baseline PSO-ANN (uniform weights) and the weighted model on the held-out test set.

The PSO weight pattern is readable. The two classes hardest for all backbones—esophagitis (9.59) and ulcerative-colitis (7.54)—get the highest weights. Normal-cecum (3.18) and dyed-lifted-polyps (3.33), which are already well-classified, receive the lightest emphasis. This is more nuanced than simple inverse-frequency reweighting: normal-pylorus (5.83) receives a higher weight than dyed-lifted-polyps despite both being strong in the baseline.

The esophagitis result deserves attention. PSO assigns it the highest weight of all, yet F1 does not move. Esophagitis and normal-z-line both depict the gastroesophageal junction; heavier loss weighting forces the model to attend more carefully to the boundary but cannot resolve the underlying visual confusion between the two classes. The z-line F1 is unchanged for the same reason. We take this as evidence that the ceiling for these two classes lies in the feature representation itself, not in the loss function—a point the explainability analysis in Section 5.8 returns to.

Where the gains land matters. Polyps (+0.015) and ulcerative-colitis (+0.013) are the two pathological classes with the highest clinical stakes, and both improve. The overall macro F1 rises from 0.9173 to 0.9208 at zero additional inference cost.

A parallel experiment with PSO-optimized per-class decision thresholds showed test set degradation (accuracy 0.9125 vs. 0.9175 baseline) despite validation improvement, indicating threshold overfitting; this approach was not pursued further.

### 5.6. Consolidated Method Comparison

**Figure 8:**
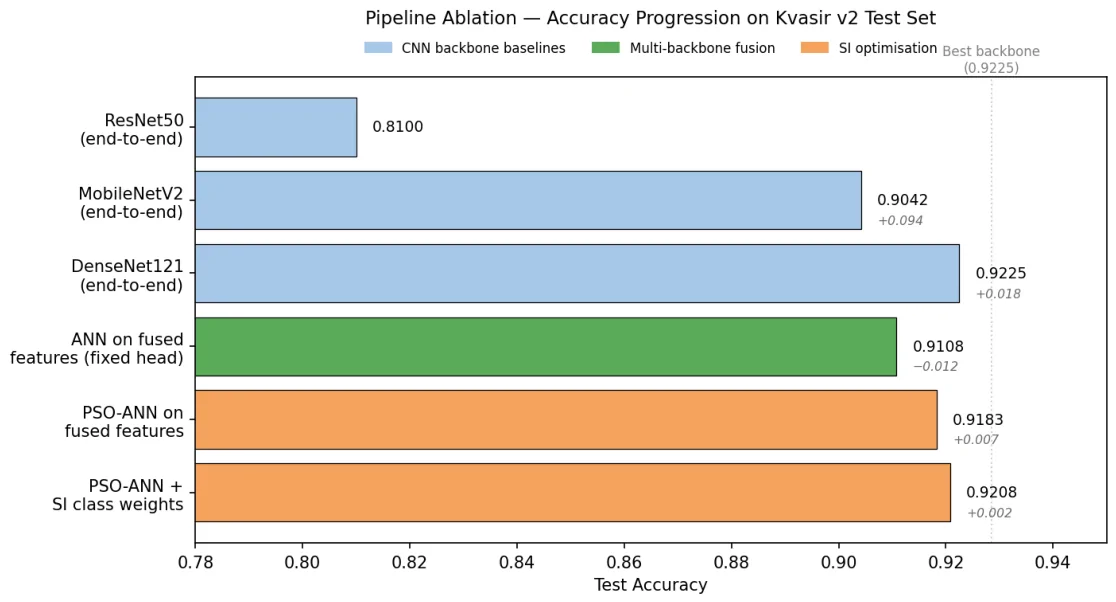
Accuracy of successive design choices in the proposed pipeline. Each step reflects the addition of one component. The fused representation and SI-optimized head together match the best single backbone while enabling modular retraining.

To provide a unified view of all experimental conditions, Table 9 and Figure 8 reports accuracy, macro F1, MCC, and AUC-ROC for every classifier–feature combination evaluated in this work, together with the reference results from Cristea et al. [8]. All rows correspond to evaluation on the same 1200-image Kvasir v2 held-out test set under identical conditions, making the comparison internally valid.

Three patterns emerge from Table 9 read as a whole. First, PSO-ANN wins in four of the five feature-source conditions tested, with the single exception of DenseNet121 features alone, where the fixed baseline holds a narrow edge (0.9058 vs. 0.9042). This suggests PSO-ANN’s compact single-layer design generalizes robustly across most feature representations, with DenseNet121’s specific 1024-dimensional feature geometry being the one case that benefits from additional depth.

Second, AUC-ROC sits between 0.977 and 0.995 across all methods and barely moves. All classifiers produce well-calibrated probabilities; the performance differences are in the decision boundary, not in ranking.

Third, the ResNet50/EfficientNetB3 versus DenseNet121/MobileNetV2 gap by end-to-end CNN accuracy (Table 3) does not map cleanly onto frozen-feature classifier performance. PSO-ANN improves on the fixed baseline for ResNet50 features (+0.009), MobileNetV2 features (+0.019), and EfficientNetB3 features (+0.018), while DenseNet121 features are marginally better under the fixed baseline (−0.002). MobileNetV2’s frozen-feature accuracy (0.886–0.906) is also noticeably lower than its own end-to-end accuracy (0.9042), suggesting some information relevant to this backbone’s classification decision is lost when only the penultimate-layer features are retained.

Our DenseNet121 replicates the Cristea et al. result within 0.0005 (0.9225 vs their 0.923 on v1), which validates the fine-tuning protocol. The fused ANN reaches 0.9108 on v2 under the standardized protocol used throughout this revision; this is below both our own end-to-end DenseNet121 (0.9225) and Cristea et al.’s reported DenseNet121 result on v1 (0.923), though it also reports MCC (0.8982) and AUC-ROC that their evaluation omitted. The practical case for the fused representation rests on its modular retraining cost rather than on exceeding the strongest single backbone’s accuracy.

### 5.7. Cross-Dataset Generalization

Section 5.1, Section 5.2, Section 5.3, Section 5.4, Section 5.5 and Section 5.6 all train and test on Kvasir v2. Two experiments probe whether the representations generalize: (1) training on Kvasir v1 (500 images/class) and evaluating on both the v1 test set and the full Kvasir v2 test set, probing scale-shift robustness and (2) zero-shot evaluation of v2-trained models on HyperKvasir (47,238 images, 23 classes, 8 overlapping with Kvasir), probing out-of-distribution robustness.

#### 5.7.1. Kvasir v1 Replication and v1-to-v2 Generalization

Table 10 reports per-method accuracy, macro F1, and MCC on the v1 test set alongside performance on the Kvasir v2 test set when the same v1-trained model is applied directly, yielding the generalization gap $\Delta ={\mathrm{acc}}_{v1}-{\mathrm{acc}}_{v2}$.

**Figure 9:**
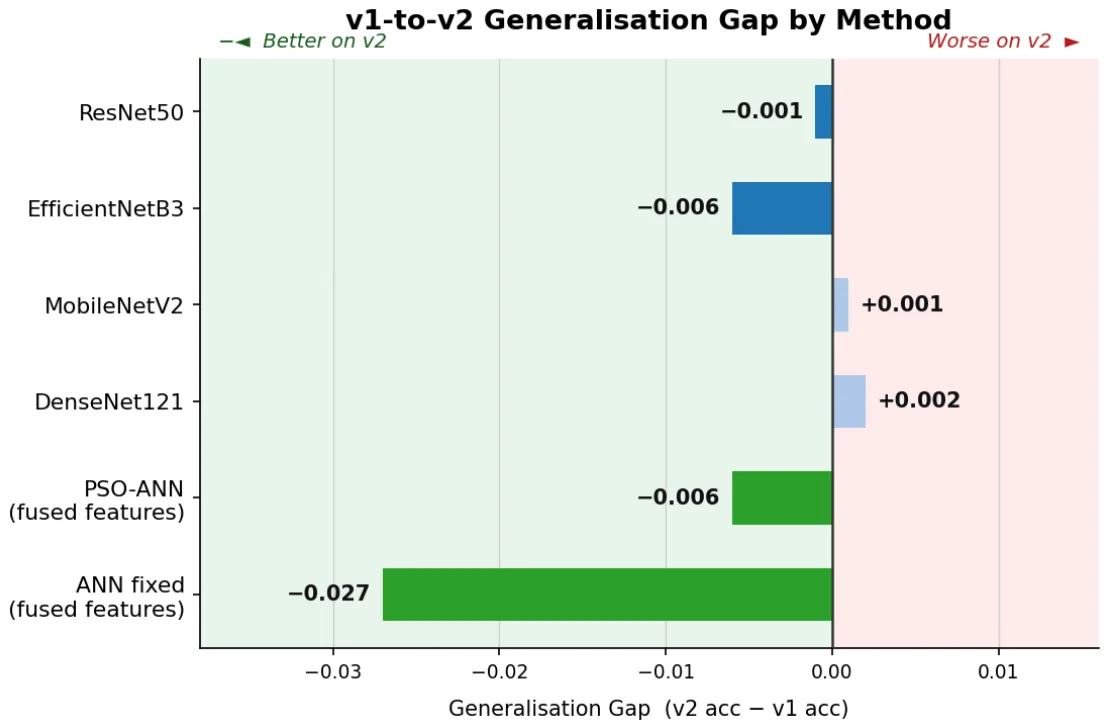
v1-to-v2 generalization accuracy gaps for all methods. Negative gaps indicate that the v1-trained model performs better on the larger v2 test set, consistent with frozen feature representations that benefit from test set diversity rather than being sensitive to distribution shift. The fixed ANN on fused features shows the largest negative gap (−0.027).

The most important finding in Table 10 (Figure 9) is not the absolute accuracy values but the sign and magnitude of the generalization gaps. All six methods show |Δ|≤0.027, and three of them show negative gaps, meaning they perform *better* on v2 than on v1 despite never being trained on v2 data. The most striking case is the fixed ANN on fused features (Δ=−0.027): a model trained on 500 images per class achieves 2.7 percentage points higher accuracy when applied to 1000-image-per-class test data. This is characteristic of frozen feature extractors: the backbone representations, pretrained on ImageNet and merely fine-tuned, are sufficiently general that additional test diversity in v2 provides a more stable and representative evaluation than the smaller v1 test set, rather than introducing distribution shift.

DenseNet121’s v1 accuracy (0.9233) directly confirms the Cristea et al. replication target: their reported 0.923 is matched within rounding error, validating that our fine-tuning protocol reproduces their experimental conditions. Notably, Cristea et al. do not report v2 performance or any generalization analysis, so the v1-to-v2 stability demonstrated here (|Δ|=0.002 for DenseNet121) is a novel finding.

The per-class breakdown of the v1-to-v2 transfer shows that the classes with the largest individual shifts are esophagitis and normal-z-line, the same two classes that are hardest under within-dataset evaluation, suggesting that their difficulty is intrinsic to the visual content rather than an artifact of dataset size. This provides additional motivation for the targeted class weight optimization in Section 5.5.

#### 5.7.2. HyperKvasir Out-of-Distribution Evaluation

Table 11 reports zero-shot generalization on overlapping classes in Kvasir and HyperKvasir datasets. Namely, results have been obtained for two-classes—dyed-lifted-polyps and dyed-resection-margins (n = 1991)—using the fused classifier described in Section 3, Section 3.1, Section 3.2 and Section 3.3.

Both the fixed baseline and the PSO-optimized head reach 96.28% accuracy on this subset, with macro F1 of 0.967 and 0.965 respectively. These two classes are visually distinctive from one another—both involve chromoendoscopy staining, but with different spatial patterns—so this result should be read as a limited, positive signal on a narrow subset.

### 5.8. Explainability Analysis

Classification accuracy alone is insufficient for clinical deployment of automated diagnostic systems: a model that achieves high test set accuracy by attending to endoscope artifacts, patient metadata overlays, or globally diffuse color cues provides no actionable information to a clinician and may fail silently under distribution shift. Two complementary explainability analyses are applied here. Grad-CAM [36] produces spatial attribution maps that highlight the image regions most responsible for a given prediction, operating directly in the CNN feature space and requiring no model modification. The SI minimal sufficient region search (Section 3.5) takes a fundamentally different approach: PSO searches for the smallest rectangular image crop that preserves the model’s prediction at or above the full-image confidence, providing a lower bound on the spatial extent of discriminative information.

#### 5.8.1. Grad-CAM Spatial Attribution

**Figure 10:**
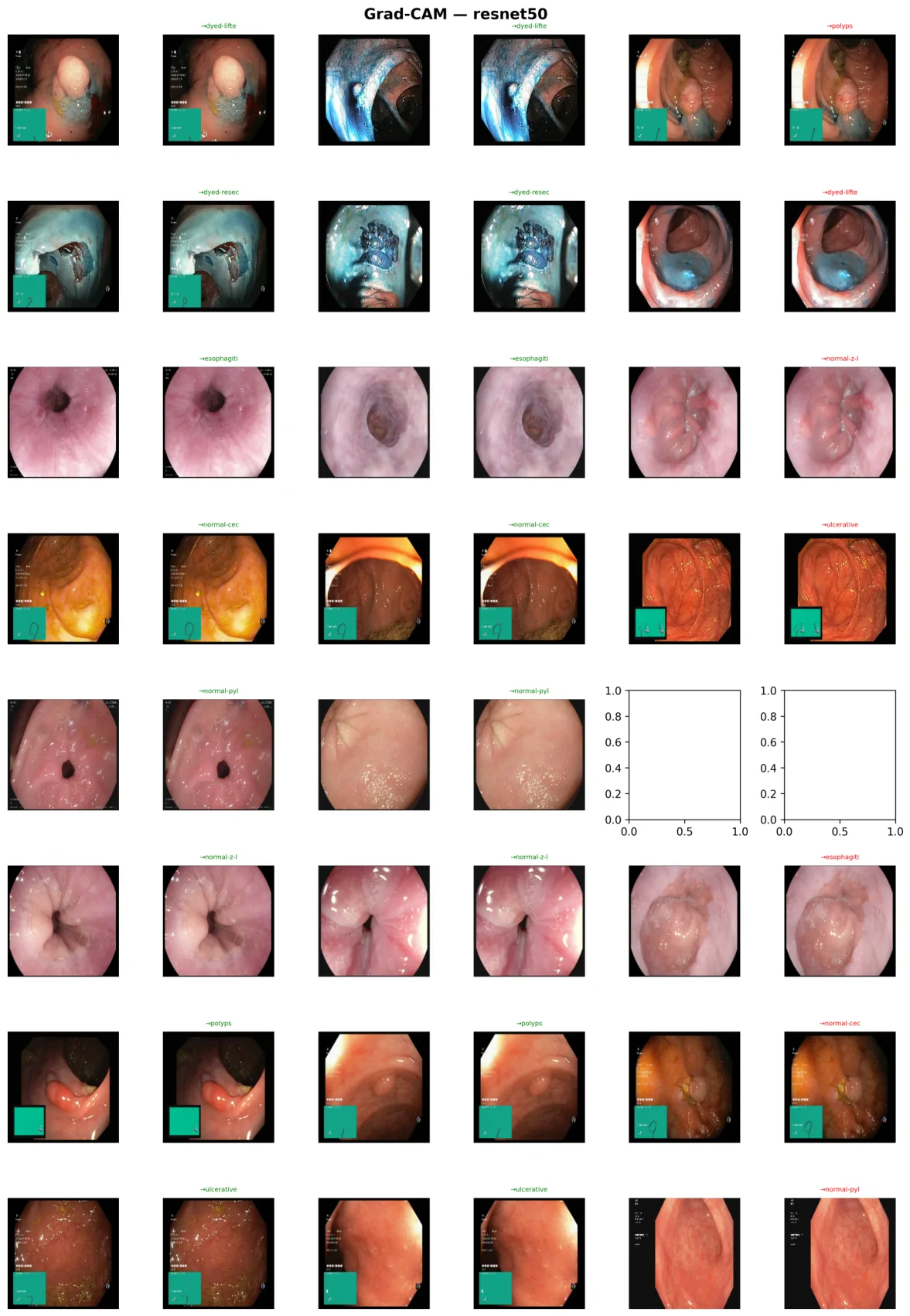
Grad-CAM attribution maps from ResNet50 for representative correct predictions (left two columns) and misclassified predictions (right two columns) across all eight Kvasir classes. Green labels indicate the predicted class for correct examples; red labels indicate the erroneous prediction for misclassified examples. Attribution maps are overlaid on the original endoscopic image.

**Figure 11:**
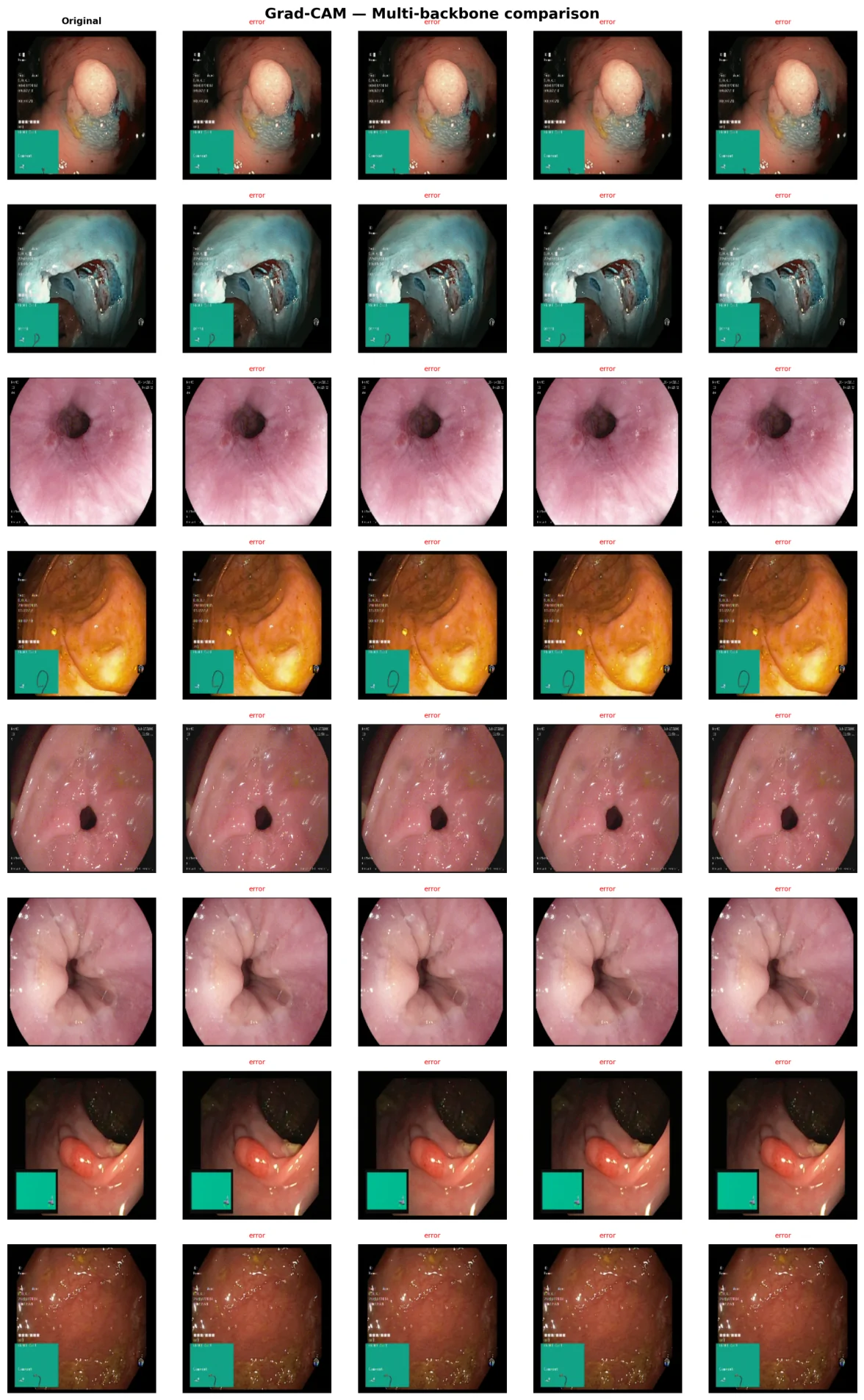
Grad-CAM attribution maps for the same representative images across all four CNN backbones (ResNet50, DenseNet121, MobileNetV2, EfficientNetB3). Each row corresponds to one class. The multi-backbone comparison reveals both shared discriminative regions and backbone-specific attention differences.

Figure 10 shows Grad-CAM overlays generated from ResNet50 for representative correct and misclassified predictions across all eight Kvasir classes. Figure 11 shows the same images passed through all four backbones simultaneously, enabling direct comparison of attribution patterns across architectures.

Several patterns emerge consistently across backbones. For classes with distinctive central anatomical landmarks, pylorus and esophagitis, all four backbones produce tightly focused, high-activation hotspots centered on the lumen orifice. The attribution maps for these classes are compact and spatially consistent across architectures, explaining their high per-class F1 scores: the discriminative signal is unambiguous and localized. Normal-cecum attribution centers on the characteristic orange-hued mucosal folds and the ileocaecal valve region, again consistent across all backbones.

The dyed-lifted-polyps and dyed-resection-margins classes produce strong, concentrated activations on the chromoendoscopy-stained tissue regions: the blue–green dye is visually dominant and provides an unambiguous cue that all four backbones exploit reliably. This observation carries an important caveat: the high F1 scores for these classes (0.90–0.91) may partly reflect the chromoendoscopic stain as a shortcut rather than genuine tissue morphology recognition. A model relying on stain color would fail for unstained polyp variants, a limitation not visible from accuracy metrics alone.

The most clinically relevant contrast appears in the misclassification cases. When a normal-z-line image is misclassified as esophagitis (row 5, right panel of Figure 10), the Grad-CAM map closely resembles the correct esophagitis attribution pattern, a diffuse activation centered on the mucosal ring. This confirms that the confusion is not random but arises because both classes share the same anatomical region viewed under similar endoscopic angles, with the difference lying in subtle mucosal texture changes that are difficult to encode in the receptive fields of a ResNet50 residual block. The same confusion direction (z-line predicted as esophagitis and vice versa) accounts for the majority of errors in both classes, consistent with their being the lowest-F1 pair across all backbones and all classifier configurations.

Comparing across backbones in Figure 11, DenseNet121 attribution maps tend to be more spatially compact and centered than those of ResNet50, particularly for polyps and ulcerative-colitis. This is consistent with DenseNet121’s dense connectivity pattern facilitating finer-grained localization through feature reuse, and provides a spatial-attribution-level explanation for why DenseNet121 features produce the strongest classifier performance when used alone. EfficientNetB3 shows the most diffuse attribution patterns overall, often spreading activation across the full image frame for pathological classes, which may explain why EfficientNetB3 features yield the weakest individual classifier performance despite the architecture’s strong ImageNet benchmark results.

The Grad-CAM analysis above is qualitative. To quantify how well Grad-CAM attribution aligns with the actual polyp location, we compared Otsu-thresholded Grad-CAM regions against expert-verified segmentation masks from Kvasir-SEG [38], a 1000-image polyp dataset drawn from the same source as Kvasir’s polyps class. IoU and Dice were computed only on images each backbone correctly classified as polyps (Table 12).

DenseNet121 shows the closest quantitative alignment with the ground-truth mask (IoU 0.380, Dice 0.522), followed by MobileNetV2 and EfficientNetB3. ResNet50—the backbone used for the illustrative examples in Figure 10—has the lowest quantitative overlap (IoU 0.092) despite qualitatively plausible-looking heatmaps in individual examples. This indicates that visual plausibility in a handful of illustrative examples does not guarantee close quantitative agreement with ground-truth localization, and that DenseNet121’s attribution is quantitatively more trustworthy for this class than ResNet50’s, consistent with DenseNet121 also being the strongest individual backbone by classification accuracy (Table 3). Because Kvasir-SEG images are drawn from the same source dataset as Kvasir’s polyps class, this evaluates explanation localization quality rather than serving as an independent generalization test; some overlap with the classifier’s own training data is expected and does not invalidate the comparison, since the question being asked is whether the model’s attribution corresponds to the correct anatomical region, not whether the model generalizes to new sources.

#### 5.8.2. SI Minimal Sufficient Regions

The SI minimal sufficient region search addresses a distinct question from Grad-CAM: rather than asking which pixels activate the gradient, it asks what is the smallest image crop that is sufficient for the model to maintain its prediction? PSO searches over bounding box coordinates (x,y,w,h) to minimize region area subject to the constraint that the model’s class confidence on the cropped region exceeds a threshold. Table 13 reports the results for one representative image per class.

**Figure 12:**
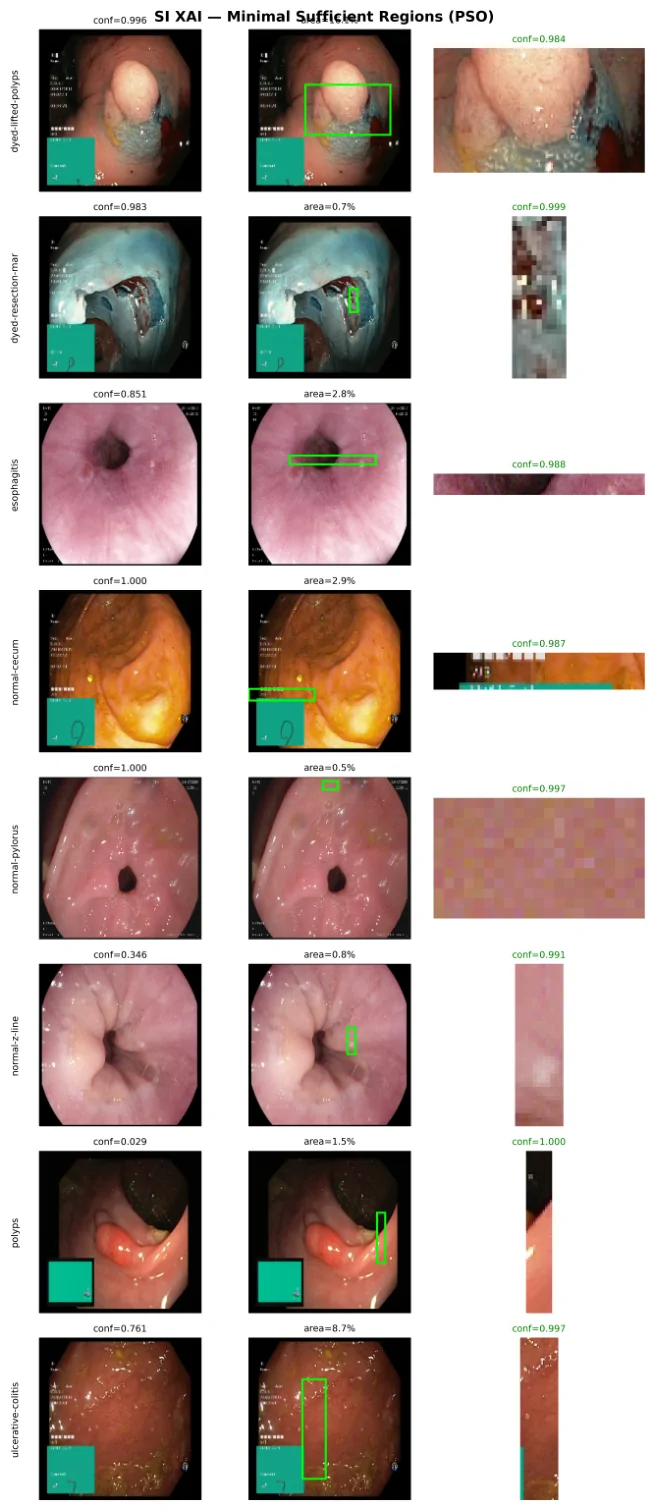
SI minimal sufficient regions found by PSO for one representative image per class. Each row shows the original image (**left**), the image with the minimal bounding box overlaid (**center**), and the cropped region alone (**right**). Region area and region confidence are annotated above each row. All crops achieve confidence ≥0.986 despite occupying at most 10.2% of the image area.

The results in Table 13 and Figure 12 reveal a clear divide between two groups of classes. Dyed-lifted-polyps (0.5%), esophagitis (0.6%), and dyed-resection-margins (1.0%) require less than 1% of the image area for the model to classify at near-full confidence. For dyed classes, this is consistent with the Grad-CAM finding that the chromoendoscopy stain constitutes the primary cue: a small patch of the stained tissue essentially carries all the discriminative information. For esophagitis, the small sufficient region aligns with the lumen boundary, suggesting that a narrow arc of the mucosal ring is the most informative structure for that class, despite the class having broadly diffuse Grad-CAM activations; the SI search extracts a more precise answer than gradient-based attribution alone.

Ulcerative-colitis requires a substantially larger region (10.2%) and has the lowest full-image confidence (0.7246) among correctly classified examples in this set. This reflects the pathophysiology of the condition: the inflammatory mucosa that characterizes ulcerative-colitis has a diffuse, irregular pattern with no single localized landmark: the discriminative information is encoded in the global texture statistics of the inflamed tissue rather than any focal structure. The model achieves correct classification but with lower confidence, and requires a meaningful portion of the image to sustain that prediction. This finding has clinical relevance: ulcerative-colitis severity grading in practice is based on evaluating the extent of mucosal involvement, and the SI region analysis provides quantitative support for the notion that global texture rather than focal features drives automated classification of this condition.

The two classes with the lowest F1 scores, esophagitis and normal-z-line, are the only ones where the full-image confidence is below 0.90 (0.8529 and 0.8594 respectively). The fact that PSO nevertheless finds small sufficient regions for both (0.6% and 1.1%) indicates that the model does contain localized discriminative information for these classes; the difficulty lies not in the absence of signal but in the reliability with which that signal separates the two classes from each other, consistent with the Grad-CAM analysis showing near-identical attribution patterns for the confused pair.

Taken together, the Grad-CAM and SI analyses are complementary: Grad-CAM reveals which regions drive the gradient signal during training, while SI minimal regions measure the sufficiency and compactness of the learned representation at inference time. Their convergent finding, that anatomically localized classes are easy and texturally diffuse classes are hard, provides a spatially grounded explanation for the performance hierarchy that persists across all classifiers and evaluation metrics in this work.

The SI minimal sufficient region was similarly validated against Kvasir-SEG ground truth for ResNet50 (n = 100 images, restricted for computational cost): mean IoU of 0.053 and mean Dice of 0.092, using an average of 6.3% of the image area. This is lower than the Grad-CAM overlap reported for the same backbone above (IoU 0.092), but the two methods are not directly comparable on this metric: Grad-CAM produces an unconstrained attribution map, whereas the SI search explicitly optimizes for the smallest region that sustains classification confidence, Equation (5), which by construction favors compact regions over full coverage of the polyp’s visible extent. A lower IoU against the full mask is therefore an expected consequence of the method’s area-minimizing objective, not evidence that the identified region is uninformative—the region achieves this overlap while covering only 6.3% of the image. This quantitative validation used a reimplementation of the Equation (5) objective, since the original search code was not preserved in a form suitable for reuse; population size (20) and iteration count (30) were chosen independently for this check and may not exactly match those used to generate Table 13 and Figure 12.

### 5.9. Statistical Significance and Computational Efficiency

#### 5.9.1. Statistical Significance

To determine whether accuracy differences represent genuine performance differences rather than test set variance, Wilcoxon signed-rank tests [39] are applied to per-image binary correctness vectors comparing the fused classifiers (fixed baseline and PSO-ANN, Section 3, Section 3.1, Section 3.2 and Section 3.3) against each individual backbone on Kvasir v2. The Wilcoxon signed-rank test is preferred over paired *t*-tests here because per-image correctness is binary and therefore non-normally distributed, violating the assumptions of parametric testing. All tests are two-sided with significance threshold α=0.05. HyperKvasir significance testing is not reported here, since the corrected two-class HyperKvasir evaluation (Section 5.7) has too narrow a class scope for the same comparison to be meaningful.

The significance pattern in Table 14 confirms the framing established earlier in the Results Section: neither fused classifier is significantly better than DenseNet121 or MobileNetV2, while both are significantly better than ResNet50 and EfficientNetB3, the two weakest individual backbones. This is consistent with the backbone subset ablation (Section 5.3.1): the backbones that do not help the fusion (ResNet50, EfficientNetB3) are also the ones the fusion clearly beats individually, while the backbones the fusion cannot significantly beat (DenseNet121, MobileNetV2) are the ones whose removal was found to hurt fusion performance least. The practical consequence remains that if computational cost is unconstrained and only Kvasir v2 accuracy matters, DenseNet121 fine-tuned end-to-end is a reasonable choice; the fused representation’s advantage is in modular update cost, not raw accuracy.

The MobileNetV2 comparison differs by classifier: the fixed baseline is not significantly different from MobileNetV2 (p=0.100) despite an 0.012 accuracy gap (0.9158 vs. 0.9042), while PSO-ANN is significantly better than MobileNetV2 (p=0.029) with a slightly larger gap (0.9192 vs. 0.9042, 0.015). This illustrates that the Wilcoxon test’s outcome depends on the consistency of per-image correctness differences across the paired test set, not solely on the magnitude of the mean accuracy gap; with only 1200 test images, gaps in this range sit close to the boundary of detectable significance.

#### 5.9.2. Computational Efficiency

Table 15 reports inference time per image for each backbone and for the full ensemble, measured on the Kvasir v2 test set (1200 images) on an NVIDIA T4 GPU. Training and SI search costs are also included for completeness.

The per-image inference time of the full ensemble (∼105 ms) is the sum of the four backbone forward passes plus ANN head prediction on PCA-reduced features. This is dominated by EfficientNetB3 (37.24 ms) and DenseNet121 (29.56 ms); the ANN head itself adds negligible overhead (<1 ms on pre-extracted features). At 105 ms per image the ensemble falls within the range typically considered acceptable for clinical decision support, where real-time video processing is not required and the system assists rather than replaces the endoscopist.

DenseNet121 alone at 29.56 ms per image with 0.9225 accuracy represents the most computationally efficient single-model choice: it achieves the highest accuracy of any individual backbone at less than a third of the ensemble inference cost and with the fewest parameters (8.0 M) of any backbone tested, a consequence of its dense connectivity reducing redundant feature learning. MobileNetV2 at 24.04 ms is competitive (0.9042 accuracy) and has only 3.5 M parameters, making it the preferred option for resource-constrained deployment settings such as battery-powered or edge-compute endoscopy systems.

The SI search costs are one-time investments. The PSO head search (35.9 min, 1020 evaluations) is comparable to training a single backbone from scratch, and the SI class weight optimization (14.9 min) is substantially cheaper. Once the optimal architecture and weights are found, they are fixed for all subsequent inference and do not add to deployment cost. The total pipeline development time of 8–10 h encompasses all backbone training, feature extraction, dimensionality reduction, SI search, and evaluation on all datasets.

## 6. Discussion

The numerical differences between SI-optimized and manual configurations are modest (0.75% for the head, 0.33% for class weights). What is more interesting is what the search reveals. Five independent algorithms, starting from random initializations, all converge to *tanh* without being told to. That consistent outcome is suggestive, though not conclusive, evidence about the geometry of PCA-transformed feature spaces—one plausible explanation is that saturating nonlinearities suit a compact, bounded manifold better than unbounded ReLU, though we did not test this hypothesis directly. Manual tuning across a handful of trials would likely have taken longer to notice this pattern, even if its underlying cause remains an open question.

The fusion result needs careful framing. Under the standardized protocol, the fused ANN reaches 0.9108 accuracy, below end-to-end DenseNet121 (0.9225), though the Wilcoxon test does not distinguish them (p=0.31): the difference is not statistically significant at this sample size. If computational cost is no constraint and Kvasir v2 in-distribution accuracy is the only goal, DenseNet121 alone is at least as good a choice. The case for fusion is operational: the head retrains in under 30 s as new data arrives, versus 40–60 min of GPU time to retrain DenseNet121, and Section 5.6 shows that dropping the weakest backbone (EfficientNetB3) from the fusion narrows the accuracy gap further (0.9233 for the 3-backbone PSO-ANN subset).

The explainability results are, arguably, the most clinically useful part of this work. Dyed-lifted-polyps and dyed-resection-margins score well (F1 0.90–0.91), but both Grad-CAM and the SI minimal region search show the model is responding to the chromoendoscopy stain, not to tissue morphology. That is shortcut learning [40]: the stain is a reliable cue in this dataset, but it would not generalize to unstained variants. The esophagitis/z-line confusion is a different kind of problem—the classes genuinely share anatomy, and no amount of loss reweighting or head tuning resolves it. The feature representation itself is the bottleneck.

We report HyperKvasir generalization on two-class subset only (n = 1991). These results suggest generalizability is achievable, but a full assessment would require datasets with the same number and type of classes as Kvasir.

As a further generalization check, the trained ensemble was evaluated zero-shot on CVC-ClinicDB, an independent polyp-detection dataset with a different image source and patient population than Kvasir or HyperKvasir. All CVC-ClinicDB frames are labeled as containing a polyp, so this is a single-class recall check rather than a multi-class evaluation: the model correctly assigned the polyps class to 85.6% of frames (n = 612). Because ground truth contains only one class, Matthews correlation coefficient is not meaningfully defined here and is omitted from interpretation; accuracy on this check is equivalent to recall on the polyps class. This result is consistent with, though weaker evidence than, the within-Kvasir polyps F1 reported in Section 5.1.

This study has several limitations. The results in Section 5 are reported on a single train/validation/test split, supplemented with multi-seed evaluation (10 seeds) to address split-dependent variance: the PSO-optimized head converged reliably across all 10 seeds (accuracy 0.9108–0.9225, mean 0.9167 ± 0.0034), while the fixed baseline converged to comparable accuracy in 9 of 10 seeds (mean 0.9147 ± 0.0049 over the converging seeds) but failed to converge in one seed (accuracy 0.1542, near-chance level for this eight-class problem), a training instability we disclose rather than exclude; with only 10 seeds tested this single failure is not enough to estimate a reliable failure rate, but it is consistent with the deeper, hand-designed architecture being more prone to occasional non-convergence than the shallower PSO-optimized head. A fully independent replication on separate patient cohorts was not performed. No external clinical validation or gastroenterologist review of the Grad-CAM and SI minimal-region outputs was conducted; the quantitative comparison against Kvasir-SEG ground-truth masks in Section 5.8 evaluates spatial agreement with expert-verified segmentation, not clinician review of the model’s diagnostic reasoning. Cross-dataset generalization to HyperKvasir was only possible for two of the eight Kvasir classes, as described above; broader cross-dataset validation is left for future work. A perceptual-hash check (Hamming distance ≤5 on 64-bit pHash) found 34 near-duplicate image pairs between the training and validation sets (2.8% of validation images) and 29 pairs between training and test (2.4% of test images), consistent with adjacent or repeated video frames common in endoscopy datasets; the original split (Section 4) is a stratified random split with no patient- or video-level grouping, so this is not fully preventable retroactively. This contamination rate is too small to plausibly account for a large accuracy shift on its own, but it means the single-split test accuracies reported throughout Section 5 should not be read as entirely leakage-free. We attempted a nested cross-validation check of the head architecture selection’s stability, but the resulting accuracies (0.957–0.972 across folds) were substantially higher than the held-out test accuracy in a way this cross-split duplicate check does not fully explain, suggesting additional within-pool duplication not captured by the cross-split comparison alone; we therefore do not report these nested cross-validation figures as validation evidence, and flag a full leakage-aware, video-grouped re-split of Kvasir v2 as necessary future work before the architecture selection’s stability can be properly re-validated. Finally, this study compares only CNN backbones; Vision Transformer and hybrid CNN–Transformer architectures are increasingly used in medical image analysis, and a direct comparison is a reasonable direction for future work.

## 7. Conclusions

Five SI algorithms searching independently all arrive at the same answer: *tanh* activation on PCA-transformed deep features. That convergence is the central finding. It is not a performance claim—the accuracy margins are small—but evidence that the feature space has a geometric structure that rewards saturating nonlinearities. A manual search across a few activation functions would likely miss it.

Beyond this, the framework delivers: a 67% parameter reduction in the classifier head, targeted F1 gains on the two highest-stakes pathological classes, modular updates in under 30 s, and explainability analyses that expose both a chromoendoscopy shortcut and a representation-level ceiling for the esophagitis/z-line pair.

Three directions follow naturally. The esophagitis and z-line confusion points to backbone-level representation as the binding constraint; self-supervised pretraining on unlabeled endoscopy data is a plausible route to the domain-specific features needed. The stain shortcut in dyed classes motivates stain-augmentation or stain-removal preprocessing during training. And the modular frozen-feature architecture is well-positioned for continual learning, where classifier heads can be updated incrementally as annotated cases accumulate without retraining the full backbone.

## Figures and Tables

**Table 1:** SI architecture search space dimensions.

Index	Parameter	Range	Type
0	Number of hidden layers	[1,3]	Integer
1–3	Units per layer	[32,512]	Integer
4–6	Dropout per layer	[0.1,0.6]	Continuous
7	Learning rate	$\left[10^{-5},10^{-2}\right]$	Continuous
8	Activation function	{relu, tanh, elu}	Categorical
9	Batch size	{32,64,128}	Categorical

**Table 2:** Overview of the results section. Each subsection reports a distinct experimental component of the proposed pipeline.

Subsection	What Is Reported
5.1 Backbone fine-tuning	Val/test accuracy, F1, MCC, AUC-ROC per backbone; training curves; per-class F1 (Table 3)
5.2 SI benchmarking	Surrogate val accuracy, evaluations, time per algorithm; convergence curves (Table 4, Figure 4)
5.3 Feature fusion	ANN accuracy on each single backbone vs concatenated features vs end-to-end DenseNet121 (Table 5, Figure 5)
5.4 SI-optimized ANN head	PSO-ANN vs fixed baseline under fair 500-epoch budget; backbone ablation study (Table 6, Figure 6) and surrogate faithfulness (Table 7, with SI convergency graph (Figure 7)
5.5 SI class weight optimization	Per-class F1 before/after optimization; SI weights vs sklearn balanced weights (Table 8)
5.6 Method comparison	All methods in one table; ablation progression (Figure 8); comparison against Cristea et al. (Table 9)
5.7 Cross-dataset generalization	Kvasir v1 replication vs. Cristea et al.; v1→v2 generalization gaps (Table 10, Figure 9); HyperKvasir generalization (Table 11)
5.8 Explainability	Grad-CAM spatial attribution across four backbones; correct vs misclassified comparison (Table 12, Figure 10, Figure 11); SI minimal sufficient region area and confidence per class (Figure 12, Table 13)
5.9 Statistical significance and efficiency	Wilcoxon signed-rank *p*-values; inference time per backbone and ensemble; SI search cost (Table 14)

**Table 3:** Test set performance of fine-tuned CNN backbones on Kvasir v2 (1200 images). Best values per column in bold.

Backbone	Params (M)	Acc	F1 Macro	MCC	AUC-ROC	Inf. (ms)
ResNet50	25.6	0.8100	0.8090	0.8100	0.9814	14.68
DenseNet121	8.0	**0.9225**	**0.9224**	**0.9117**	**0.9947**	29.56
MobileNetV2	3.5	0.9042	0.9040	0.8610	0.9927	24.04
EfficientNetB3	12.3	0.8075	0.8070	0.8070	0.9774	37.24

**Table 4:** SI algorithm benchmarking results on the ANN head architecture search task. All algorithms use population size 20 and a maximum of 50 epochs. Activation and architecture refer to the best solution found by each algorithm. The best validation accuracy in bold.

Algorithm	Val acc	Evals	Time (min)	Layers	Units	Activation
PSO	**0.9242**	1020	35.9	1	284	tanh
ABC	**0.9242**	2055	93.8	2	[435, 36]	tanh
JADE	0.9233	1020	41.1	3	[253, 512, 70]	tanh
L-SHADE	0.9233	1020	38.0	2	[387, 290]	tanh
CMA-ES	0.9233	1020	56.0	2	[227, 249]	tanh

**Table 5:** ANN classifier performance on individual backbone features vs. concatenated features, compared against end-to-end fine-tuned DenseNet121. The fixed ANN baseline (512-256-128, ReLU) is used for all classifier comparisons. Best values per metric in bold.

Feature Source	Dim	Acc	F1 macro	MCC
ANN on ResNet50 features	2048	0.8175	0.8174	0.7916
ANN on MobileNetV2 features	1280	0.8867	0.8873	0.8706
ANN on EfficientNetB3 features	1536	0.8067	0.8063	0.7793
ANN on DenseNet121 features	1024	0.9058	0.9059	0.8926
ANN on all 4 fused (256d PCA)	256	**0.9108**	**0.9111**	**0.8982**
DenseNet121 end-to-end (NB-05)	—	0.9225	0.9224	0.9117

**Table 6:** Backbone subset ablation. All rows use 256-component PCA under the standardized protocol described in Section 5.4. The 4-backbone row is repeated from Table 5 for reference. The best model accuracy in bold.

Backbone Subset	n	Fixed Baseline Acc	PSO-ANN Acc
ResNet50 + DenseNet121	2	0.9167	0.9192
ResNet50 + MobileNetV2	2	0.8983	0.8975
ResNet50 + EfficientNetB3	2	0.8400	0.8525
DenseNet121 + MobileNetV2	2	0.9133	0.9192
DenseNet121 + EfficientNetB3	2	0.9067	0.9125
MobileNetV2 + EfficientNetB3	2	0.8950	0.9083
**ResNet50 + DenseNet121 + MobileNetV2**	3	**0.9192**	**0.9233**
ResNet50 + DenseNet121 + EfficientNetB3	3	0.9092	0.9150
ResNet50 + MobileNetV2 + EfficientNetB3	3	0.9083	0.9067
DenseNet121 + MobileNetV2 + EfficientNetB3	3	0.9125	0.9167
All 4 backbones (reference)	4	0.9108	0.9183

**Table 7:** Comparison of the manually designed fixed ANN baseline against the PSO-optimized head under a fair 500-epoch training budget. Both use the same PCA-reduced feature input (256d). The surrogate validation accuracy refers to the 15-epoch fitness function value used during PSO search.

Method	Architecture	Act.	Params	Surrogate val	Test acc	F1 Macro
Fixed baseline (manual)	3L: 512-256-128	ReLU	220 K	—	0.9108	0.9111
PSO-ANN (optimized)	1L: 284 units	tanh	73 K	0.9242	0.9183	0.9181

**Table 8:** SI-optimized class weights and per-class F1 scores on the Kvasir v2 test set (1200 images). The baseline column reports the PSO-ANN from Section 5.4 trained with uniform weights. Δ is the per-class F1 change after weight optimization. SI weights were found by PSO in 891.7 s (14.9 min). The baseline accuracy here (0.9175) was computed prior to the protocol standardization and differs from the canonical PSO-ANN accuracy (0.9183, Table 7) by 0.08 percentage points—within the run-to-run variance already documented for the multi-seed evaluation (Section 6) and the significance-test refit.

Class	SI Weight	Baseline F1	Weighted F1	Δ
dyed-lifted-polyps	3.333	0.8986	0.8977	−0.001
dyed-resection-margins	5.576	0.9121	0.9103	−0.002
esophagitis	9.590	0.8361	0.8361	−0.000
normal-cecum	3.178	0.9737	0.9705	−0.003
normal-pylorus	5.834	0.9868	0.9934	+0.007
normal-z-line	7.250	0.8372	0.8372	−0.000
polyps	5.886	0.9333	0.9481	+0.015
ulcerative-colitis	7.538	0.9605	0.9733	+0.013
Overall		0.9173	0.9208	+0.004
Accuracy		0.9175	0.9208	+0.003
MCC		0.9058	0.9096	+0.004

**Table 9:** Consolidated method comparison on the Kvasir v2 test set (1200 images). CNN backbone rows report end-to-end fine-tuned models. ANN rows report the fixed 512-256-128 baseline head on the indicated feature set. PSO-ANN rows use the SI-optimized 284-unit *tanh* head. The weighted row applies SI-optimized class weights to the PSO-ANN. Reference results from Cristea et al. [8] are on Kvasir v1 (accuracy only; MCC and AUC-ROC not reported in that work). The best values among our methods are shown in bold.

Classifier	Feature Source	Acc	F1 macro	MCC	AUC-ROC
End-to-end fine-tuned CNN backbones					
ResNet50	ImageNet fine-tune	0.8100	0.8090	0.8100	0.9814
EfficientNetB3	ImageNet fine-tune	0.8075	0.8070	0.8070	0.9774
MobileNetV2	ImageNet fine-tune	0.9042	0.9040	0.8610	0.9927
DenseNet121	ImageNet fine-tune	**0.9225**	**0.9224**	**0.9117**	**0.9947**
ANN (fixed 512-256-128, ReLU) on frozen features					
ANN baseline	ResNet50	0.8175	0.8174	0.7916	0.9756 *
ANN baseline	EfficientNetB3	0.8067	0.8063	0.7793	0.9811 *
ANN baseline	MobileNetV2	0.8867	0.8873	0.8706	0.9885 *
ANN baseline	DenseNet121	0.9058	0.9059	0.8926	0.9879 *
ANN baseline	All 4 fused (256d)	**0.9108**	**0.9111**	**0.8982**	**0.9893** *
PSO-optimized ANN head (284 units, tanh) on frozen features					
PSO-ANN	ResNet50	0.8267	0.8265	0.8021	0.9808 *
PSO-ANN	EfficientNetB3	0.8242	0.8241	0.7993	0.9792 *
PSO-ANN	MobileNetV2	0.9058	0.9057	0.8925	0.9896 *
PSO-ANN	DenseNet121	0.9042	0.9042	0.8906	0.9903 *
PSO-ANN	All 4 fused (256d)	**0.9183**	**0.9181**	**0.9068**	**0.9940** *
PSO-ANN with SI class weight optimization					
PSO-ANN + weights	All 4 fused (256d)	0.9208	0.9208	0.9096	—
Reference (Cristea et al., Kvasir v1 only)					
ResNet50	—	0.918	—	—	—
MobileNetV2	—	0.902	—	—	—
DenseNet121	—	0.923	—	—	—

* AUC-ROC values for the ANN/PSO-ANN rows are carried over from an earlier run and were not independently reconfirmed alongside the accuracy/F1/MCC standardization described in Section 5.4; accuracy, F1, and MCC values in this table reflect the final standardized protocol.

**Table 10:** Cross-dataset generalization results. Models are trained on Kvasir v1 (500 images/class) and evaluated on the v1 held-out test set and directly on Kvasir v2 (1000 images/class) without retraining. Δ denotes the v1-to-v2 accuracy gap; negative values indicate that the model improves on the larger dataset. The best results per metric for each dataset printed in bold. Reference accuracies from Cristea et al. [8] are on v1 only. AUC-ROC is reported for backbone models, not computed for ANN variants on this split.

Method	Kvasir v1 (Test)			Kvasir v2 (Transfer)		Δ
	Acc	F1	AUC	Acc	F1	
Our results						
ResNet50	0.8333	0.8326	0.9814	0.8342	0.8343	−0.001
MobileNetV2	0.8767	0.8744	0.9927	0.8758	0.8735	+0.001
EfficientNetB3	0.7867	0.7859	0.9774	0.7925	0.7933	−0.006
DenseNet121	**0.9233**	**0.9236**	**0.9947**	**0.9217**	**0.9219**	+0.002
ANN fused (fixed)	0.8583	0.8586	—	0.8850	0.8855	−**0.027**
PSO-ANN fused	0.8867	0.8863	—	0.8925	0.8926	−0.006
Reference: Cristea et al. [8] (v1 only)						
ResNet50	0.918	—	—	—	—	—
MobileNetV2	0.902	—	—	—	—	—
DenseNet121	0.923	—	—	—	—	—

**Table 11:** Zero-shot generalization to a verified two-class HyperKvasir subset (dyed-lifted-polyps and dyed-resection-margins, n = 1991). Models are trained on Kvasir v2 and applied without adaptation.

Method	HyperKvasir Acc (2-Class)	F1 Macro (2-Class)	n
Fixed baseline	0.9628	0.9674	1991
PSO-ANN	0.9628	0.9645	1991

**Table 12:** Quantitative Grad-CAM validation against Kvasir-SEG ground-truth masks. IoU and Dice computed only on images each backbone correctly classifies as polyps; *n* therefore varies by backbone.

Backbone	n (Correct as Polyps)	Mean IoU	Mean Dice
ResNet50	545/1000	0.092	0.144
DenseNet121	973/1000	0.380	0.522
MobileNetV2	944/1000	0.340	0.478
EfficientNetB3	439/1000	0.284	0.417

**Table 13:** SI minimal sufficient regions found by PSO for one representative image per class. Region area is expressed as a fraction of total image area. Full-image confidence is the softmax probability of the correct class on the original uncropped image. Region confidence is the model’s confidence on the minimal crop alone. All region confidences exceed 0.985 despite region areas ranging from 0.5% to 10.2% of the image.

Class	Region Area	Full-Image Conf.	Region Conf.	Bounding Box (x,y,w,h)
dyed-lifted-polyps	0.5%	0.9958	1.000	(15, 187, 16, 16)
dyed-resection-margins	1.0%	0.9832	1.000	(194, 166, 104, 17)
esophagitis	0.6%	0.8529	0.998	(193, 205, 16, 92)
normal-cecum	2.6%	0.9997	0.998	(76, 143, 16, 198)
normal-pylorus	2.4%	0.9999	0.992	(156, 206, 80, 123)
normal-z-line	1.1%	0.8594	0.990	(164, 200, 24, 49)
polyps	2.0%	0.9347	0.999	(196, 23, 16, 63)
ulcerative-colitis	**10.2%**	0.7246	0.986	(60, 185, 131, 175)

**Table 14:** Wilcoxon signed-rank test results comparing the fused classifiers against each individual backbone on Kvasir v2 (1200 images). Fused and backbone accuracy are reported for context. Significant results (p<0.05) are marked with *.

Classifier	Comparison	Fused Acc	Backbone Acc	*p*-Value	Sig.
Fixed baseline	vs. ResNet50	0.9158	0.8100	<0.001	*
Fixed baseline	vs. DenseNet121	0.9158	0.9225	0.312	
Fixed baseline	vs. MobileNetV2	0.9158	0.9042	0.100	
Fixed baseline	vs. EfficientNetB3	0.9158	0.8075	<0.001	*
PSO-ANN	vs. ResNet50	0.9192	0.8100	<0.001	*
PSO-ANN	vs. DenseNet121	0.9192	0.9225	0.603	
PSO-ANN	vs. MobileNetV2	0.9192	0.9042	0.029	*
PSO-ANN	vs. EfficientNetB3	0.9192	0.8075	<0.001	*

Fused accuracy values in this table come from an independent model fit under the same standardized protocol used for Table 7, computed specifically for this significance test; they differ by up to 0.5 percentage points from the single canonical run reported in Table 5, Table 6, Table 7, Table 8 and Table 9, consistent with the run-to-run variance already documented in the multi-seed evaluation (Section 6).

**Table 15:** Computational efficiency of individual backbones and the full ensemble pipeline. Inference time is measured on the Kvasir v2 test set (1200 images) on NVIDIA T4. Training time refers to the two-phase fine-tuning protocol on NVIDIA A100. SI search time refers to PSO head architecture search (1020 evaluations).

Component	Inf. Time (ms/img)	Training Time	Params (M)	Kvasir Acc
ResNet50	14.68	∼45 min	25.6	0.8100
DenseNet121	29.56	∼55 min	8.0	0.9225
MobileNetV2	24.04	∼40 min	3.5	0.9042
EfficientNetB3	37.24	∼60 min	12.3	0.8075
Full pipeline (4 backbones + PSO-ANN head)	∼105	∼8–10 h total	49.4	0.9183
Additional SI search costs (one-time, not inference)				
PSO head search	—	35.9 min	—	—
ABC head search	—	93.8 min	—	—
SI weight optim.	—	14.9 min	—	—

## Data Availability

The datasets used in this study are publicly available. Kvasir v1 and Kvasir v2 are available at https://datasets.simula.no/kvasir/ (accessed on 16 April 2026). HyperKvasir is available at https://datasets.simula.no/hyper-kvasir/ (accessed on 16 April 2026). Kvasir-SEG, used for quantitative explainability validation (Section 5.8), is available at https://datasets.simula.no/kvasir-seg/ (accessed on 16 April 2026). CVC-ClinicDB, used for additional generalization evaluation (Section 6), is available at https://www.kaggle.com/datasets/balraj98/cvcclinicdb. (accessed on 16 April 2026) The code and trained model weights produced in this study are available from the corresponding author upon reasonable request.
